# Supplementary material for: Climate policy support as a tool to control others’ (but not own) environmental behavior?
Source: PLoS One. 2022 Jun 22;17(6):e0269030. doi: 10.1371/journal.pone.0269030 (PMC9216538; doi:10.1371/journal.pone.0269030)
Supplement: Attachment — Submitted filename: 22MNA-CC-HB.pdf [file pone.0269030.s001.pdf]

Cambridge Elements
Applied Social Psychology

Climate Change and Human Behavior

Andreas Miles-Novelo and Craig A. Anderson

Downloaded from https://www.cambridge.org/core. Iowa State University Library, on 07 Feb 2022 at 18:20:25, subject to the Cambridge Core terms of use, available at https://www.cambridge.org/core/terms. https://doi.org/10.1017/9781108953078

ISSN 2631-777X (online)
ISSN 2631-7761 (print)

Downloaded from https://www.cambridge.org/core. Iowa State University Library, on 07 Feb 2022 at 18:20:25, subject to the Cambridge Core terms of use, available at https://www.cambridge.org/core/terms. https://doi.org/10.1017/9781108953078

Cambridge Elements

Elements in Applied Social Psychology
edited by
Susan Clayton
College of Wooster, Ohio

CLIMATE CHANGE AND HUMAN BEHAVIOR

Impacts of a Rapidly Changing Climate on Human Aggression and Violence

Andreas Miles-Novelo
Iowa State University
Craig A. Anderson
Iowa State University

CAMBRIDGE
UNIVERSITY PRESS

Downloaded from https://www.cambridge.org/core. Iowa State University Library, on 07 Feb 2022 at 18:20:25, subject to the Cambridge Core terms of use, available at https://www.cambridge.org/core/terms. https://doi.org/10.1017/9781108953078

CAMBRIDGE
UNIVERSITY PRESS

University Printing House, Cambridge CB2 8BS, United Kingdom

One Liberty Plaza, 20th Floor, New York, NY 10006, USA

477 Williamstown Road, Port Melbourne, VIC 3207, Australia

314–321, 3rd Floor, Plot 3, Splendor Forum, Jasola District Centre,
New Delhi – 110025, India

103 Penang Road, #05–06/07, Visioncrest Commercial, Singapore 238467

Cambridge University Press is part of the University of Cambridge.

It furthers the University's mission by disseminating knowledge in the pursuit of education, learning, and research at the highest international levels of excellence.

www.cambridge.org
Information on this title: www.cambridge.org/9781108948678
DOI: 10.1017/9781108953078

© Andreas Miles-Novelo and Craig A. Anderson 2022

This publication is in copyright. Subject to statutory exception and to the provisions of relevant collective licensing agreements, no reproduction of any part may take place without the written permission of Cambridge University Press.

First published 2022

A catalogue record for this publication is available from the British Library.

ISBN 978-1-108-94867-8 Paperback
ISSN 2631-777X (online)
ISSN 2631-7761 (print)

Cambridge University Press has no responsibility for the persistence or accuracy of URLs for external or third-party internet websites referred to in this publication and does not guarantee that any content on such websites is, or will remain, accurate or appropriate.

Downloaded from https://www.cambridge.org/core. Iowa State University Library, on 07 Feb 2022 at 18:20:25, subject to the Cambridge Core terms of use, available at https://www.cambridge.org/core/terms. https://doi.org/10.1017/9781108953078

Climate Change and Human Behavior

Impacts of a Rapidly Changing Climate on Human Aggression and Violence

Elements in Applied Social Psychology

DOI: 10.1017/9781108953078
First published online: February 2022

Andreas Miles-Novelo
Iowa State University

Craig A. Anderson
Iowa State University

Author for correspondence: Andreas Miles-Novelo, andreasm@iastate.edu

Abstract: Much of the current rhetoric surrounding climate change focuses on the physical changes to the environment and the resulting material damage to infrastructure and resources. Although there has been some dialogue about secondary effects (namely, mass migration), little effort has been given to understanding how rapid climate change is affecting people on group and individual levels. In this Element, we examine the psychological impacts of climate change, focusing especially on how it will lead to increases in aggressive behaviors and violent conflict, and how it will influence other aspects of human behavior. We also look at previously established psychological effects and use them to help explain changes in human behavior resulting from rapid climate change, as well as to propose actions that can be taken to reduce climate change itself and mitigate harmful effects on humans.

Keywords: climate change, psychology, violence, aggression, conflict

© Andreas Miles-Novelo and Craig A. Anderson 2022

ISBNs: 9781108948678 (PB), 9781108953078 (OC)
ISSNs: 2631-777X (online), 2631-7761 (print)

Downloaded from https://www.cambridge.org/core. Iowa State University Library, on 07 Feb 2022 at 18:20:25, subject to the Cambridge Core terms of use, available at https://www.cambridge.org/core/terms. https://doi.org/10.1017/9781108953078

Contents

1 Introduction 1

2 The Climate Change–Violence Model 4

3 The Direct Effects of Heat On Cognition and Behavior: Route 1 8

4 Indirect Effects of Climate Change On Aggression and Violence 19

5 What Solutions Are There? 38

6 Conclusions 45

References 47

Downloaded from https://www.cambridge.org/core. Iowa State University Library, on 07 Feb 2022 at 18:20:25, subject to the Cambridge Core terms of use, available at https://www.cambridge.org/core/terms. https://doi.org/10.1017/9781108953078

1 Introduction

Although one may be inclined to view climate change as the impending demolition of Earth, we eschew such a pessimistic view. Instead, we adopt the view espoused by the famous British science-fiction author Douglas Adams and advise everyone, “don’t panic.” Although Adams would suggest that we each carry a towel to help combat our individual and collective anxiety, there is perhaps a more approachable alternative for those of us looking for hope – and that is to become informed of the problem(s) and potential solutions at hand and to spread awareness of these to others. A later section of this Element outlines how human psychology can be used to combat climate change and its impending threats, but for now, readers should note that no matter how dire or urgent the circumstances may seem (and they are, indeed, both dire *and* urgent), remember that even though human nature has gotten us here, human ingenuity can help heal our planet while making it safer and more humane for its inhabitants (Homo sapiens and other species alike).

First, we highlight the urgency with which humans must act. We devote little time explaining *why* or *how* scientists know that rapid global warming is indeed happening, that it is man-made, and that the consequences are dire. Indeed, spending time defending the science of climate change may be part of why people, in general, haven’t gotten over the hurdle of fully embracing rapid climate change as an existential threat. Social psychology has routinely shown that even if people can view an observable truth, if there is any attention given to the denial of it, or if there is a collective action to ignore or alter the truth, then humans will do so. A famous social psychology study (Asch, 1952/1972) looked at undergraduates giving feedback on the length of lines projected in a classroom. Asch demonstrated that by having all but one of the undergraduates in the room (i.e., confederates of the experimenter as “fake students” and one “real” student who was the participant) suggest that they thought a line that was clearly shorter than other lines in a group was not, many real participants would side with the incorrect but overwhelming majority. Similarly, much of social psychology has been devoted to studying the whys and hows of getting people to behave in ways that contradict their own beliefs and values and how group pressure and misinformation can distort perception and action in the most extreme ways (e.g., the Holocaust).

Understanding these basic effects of group dynamics makes it particularly alarming to learn of the amount of attention that climate change denialists receive. A recent study in *Nature Communications* reported that prominent climate change denialists received almost 50 percent *more* media coverage than top-tier climate scientists (Peterson, Vincent, & Westerling, 2019).

Downloaded from https://www.cambridge.org/core. Iowa State University Library, on 07 Feb 2022 at 18:20:25, subject to the Cambridge Core terms of use, available at https://www.cambridge.org/core/terms. https://doi.org/10.1017/9781108953078

Although one may be swayed by a surface level defense of "well, both sides of an argument should receive equal representation," most thoughtful people (including some at the British Broadcasting Corporation) would agree that public news presentations of factual information: 1) should not give equal time to science denialists and conspiracy theorists as is given to actual expert climate scientists; and 2) the true scientific experts should be given much more media time and space but, further, 3) that this is not an issue of "both sides." Climate change is happening, and people (and governments) must act decisively and assertively to adequately address this crisis. Denial of not only climate change, but also of its severity and urgency, is simply a denial of the scientific truth; it is not a "different perspective" or a different interpretation of it. Psychological research has found that by positioning a claim that is false (such as climate change denialists do) with any sort of validity makes people more inclined to believe that there could be truth to the statements, even if this is later corrected by scientific fact. Indeed, even a purely hypothetical theory about how the world works, that is, a theory that the person knows they made up as part of a thought exercise, becomes resistant to change (e.g., Anderson & Kellam, 1992).

The immediacy of the threat of climate change cannot be overstated. One common threshold that scientists and most governments worldwide agreed on is the need to keep the global temperature rise under 1.5°C/2.7°F, and that to do so, we would need to reduce carbon emissions by 45–50 percent by 2030 and to achieve "net zero" emissions by 2050 (see Kyoto and Paris accords). However, not only do governing bodies keep missing these carbon emissions targets, they are, instead, blowing by them. Recent projections have noted that we have *already* failed to take the appropriate political and societal steps to achieve this goal (Pielke, 2019). New targets have been set, with many experts pleading to keep the temperature increase under 2 or 3°C (3.6/5.4°F). However, there is reason to believe that these goals may now be unattainable.

A recent report from David Spratt and Ian Dunlop (2018) details the possible outcome of this clear failure to meet the goals set by previous political gatherings. In their report, they detail how the 2015 *Paris Agreement*, while ambitious and productive, failed to account for all the environmental factors at play. They note that:

> With the commitments by nations to the 2015 Paris Agreement, the current path of warming is 3°C or more by 2100. But this figure does not include "long-term" carbon-cycle feedbacks, which are materially relevant now and in the near future due to the unprecedented rate at which human activity is perturbing the climate system. Taking these into account, the Paris path would lead to around 5°C of warming by 2100. (p. 6)

Downloaded from https://www.cambridge.org/core. Iowa State University Library, on 07 Feb 2022 at 18:20:25, subject to the Cambridge Core terms of use, available at https://www.cambridge.org/core/terms. https://doi.org/10.1017/9781108953078

A 5°C/9°F increase is something beyond any current mainstream rhetoric from nonscientists. Even if humans keep the temperature increase below 5°C/9° F, existential concern for the survival of our species starts well before then. The World Bank noted that a 4°C/7.2°F increase would be "beyond adaptation" (Spratt & Dunlop, 2018; Spratt, Dunlop & Barrie, 2019). More terrifying, as Spratt et al., 2019 note, is the idea of a runaway "hothouse Earth scenario," which they say could start at 2°C or even lower.

Spratt et al., 2019 describe this "hothouse Earth scenario" as "system feedbacks and their mutual interaction could drive the Earth system climate to a point of no return, whereby further warming would become self-sustaining." At this juncture, there is nothing we as humans could do to stop the temperature from climbing ever higher and for the planet to degrade past the point of sustainability.

So, what is being done to curb this horrifying reality? Most recently, American President Joe Biden noted in his climate plan a goal to invest almost $2 trillion dollars into cutting all carbon emissions by 2050. However, if the above scenario and projections are to be believed, cutting emissions by 2050 will simply be too late. Spratt et al., 2019 note that, at our current pace, we could reach a 1.6°C/2.9°F increase in global temperature by 2030 and that by 2050 we could already surpass 2.4°C, with a high likelihood of already reaching 3–4°C by 2050.

If this happens, the impact on human civilization would be devastating. Spratt et al., 2019 outline the likely consequences on Earth in 2050 and beyond. By 2050 sea-level rise would already surpass 0.5 meters (1.64 feet); by 2100, the sea-level rise would reach at least two to three meters, and possibly as high as twenty-five meters. By 2050, 35 percent of the land surface on the globe will be subjected to twenty or more days of "lethal heat conditions, beyond the threshold for human survivability"; this would affect more than 55 percent of the global population. Both the Jet Stream and the Gulf Stream would become severely destabilized, throwing off weather patterns necessary for basic ecological systems in Europe and Asia. North America would continue to experience increases in devastating droughts, wildfires, and other environmental disasters. Mexico and Central America would see annual rainfall decreases of 50 percent, allowing for semi-permanent El Niño conditions. Arctic ice would be all but gone, the Amazon region completely decimated, and coral reefs extinct. Water shortages would become the new normal, and large swaths of current tropical climates would become unfarmable and uninhabitable.

This all goes without saying that even if humans miraculously put a stopper in the drastic increase in global temperature, irreversible and incomprehensible damage has already been done. Even if we keep warming to around 2°C/3.6°F,

Downloaded from https://www.cambridge.org/core. Iowa State University Library, on 07 Feb 2022 at 18:20:25, subject to the Cambridge Core terms of use, available at https://www.cambridge.org/core/terms. https://doi.org/10.1017/9781108953078

more than 1 billion people could become displaced (Miles-Novelo & Anderson, 2019; Spratt et al., 2019; Wariaro & Hoopert, 2018). Droughts, wildfires, water shortages, crop failures, and severe tropical weather events are already on the rise and becoming more frequent and extreme. We already have millions of species, including both plants and animals, on the brink of extinction, and many of our essential ecosystems are in extreme danger.

Even if we can save our planet, and our species, from the most extreme threats of rapid climate change, it cannot be done without radical changes to our society and to our psychology. These sudden climate changes are going to rapidly alter the way humans behave and interact with others. It will affect cognitive, emotion, and decision-making systems and put many countries in politically dangerous situations requiring the accommodation of massive numbers of displaced people.

2 The Climate Change–Violence Model

One important question for behavioral scientists is this: Can we predict, explain, and modify important changes in behaviors concerning the climate change crisis? Further, can the behavioral sciences be usefully employed to combat both climate change and the concomitant harmful effects on human behavior?

The Climate Change–Violence Model (see Figure 1; also refer to Miles-Novelo & Anderson, 2019)[1] highlights how rapid climate change would

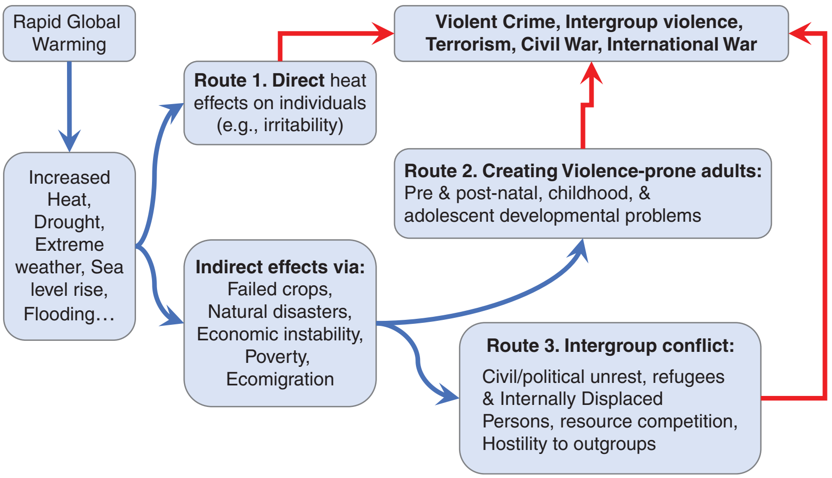

Figure 1 How rapid climate change increases violence

[1] Our team first addressed these issues in Anderson & DeLisi, 2011.

Downloaded from https://www.cambridge.org/core. Iowa State University Library, on 07 Feb 2022 at 18:20:25, subject to the Cambridge Core terms of use, available at https://www.cambridge.org/core/terms. https://doi.org/10.1017/9781108953078

influence human aggression and violence. The model demonstrates that there are three major pathways through which rapid climate change will increase human aggression and violence through one direct and two indirect mechanisms. The direct mechanism is known as "the heat effect." This is the well-replicated finding that as people become "uncomfortably" hot, they become more irritable, perceive other people's behavior as more threatening and aggressive, think more aggressively, and behave more violently (Anderson, 2001).

The indirect mechanisms (from things such as failed crops, water shortages, mass migration, and political instability) stem from (a) developmental factors that will increase the likelihood of children becoming violence-prone adolescents and adults (e.g., malnourishment), and (b) factors that increase intergroup conflicts (e.g., mass migration).

In this Element, we broaden the model in beyond human aggression/violence, although that remains the most important behavioral impact. Rapid global warming affects behavior through three pathways: a direct path (how the environment affects individuals), a developmental path (water and food shortages, growing up in disaster-ridden areas), and a group-level path (fighting over resources, the acceptance or rejection of migrants). Using this framework, we consider previously established findings from psychology, as well as other fields such as anthropology, history, political science, and sociology to demonstrate the massive change humans will experience. We also use past research to show how societies can take effective and drastic action in the immediate future, as well as how to change people's attitudes about climate change, increase awareness of the threat that our species faces, and increase effective behaviors that reduce speed and amount of global warming, and decrease the expected surge in aggressive and violent behavior.

Definitions and Overview of Aggression and Violence

As behavioral scientists, we want to make sure that readers understand the scientific meanings of the terms we use, as they can be much more specific than the general public's use of these terms. We define "aggression" as *behavior that is intended to harm another human who wishes to avoid that harm*. This can come in many forms, including physical (such as punching someone), verbal (shouting a racial slur at someone), or relational (spreading false rumors about someone). "Violence" is defined as a *severe form of physical aggression*, behaviors that, if successfully completed, are likely to necessitate medical attention. Psychologists view aggressive behaviors as existing on a continuum, with violence being at the most extreme end of the aggression continuum. Decades of research support this continuum view, which benefits

Downloaded from https://www.cambridge.org/core. Iowa State University Library, on 07 Feb 2022 at 18:20:25, subject to the Cambridge Core terms of use, available at https://www.cambridge.org/core/terms. https://doi.org/10.1017/9781108953078

from the ability to extract reasonably accurate predictions about violence from studies of less extreme forms of aggression.

Direct and Indirect Pathways

Our original model of climate change's effect on aggression and violence identified mechanisms by which rapid climate change will make people (and groups of people) behave violently and showed how those mechanisms interact with one another. It is helpful to recognize that many factors affect the likelihood of behaving aggressively or violently. There are hundreds (maybe thousands) of variables that influence how people behave in any given way in any given context. Psychologists often attempt to identify variables that have the biggest impact on specific harmful behaviors, variables called "risk factors." "Risk factors" in psychology are typically those variables known to increase the odds of someone acting in a particular harmful manner, in this case, those that increase aggression and violence. A thorough knowledge of risk factors for violence and of the likely consequences of rapid climate change on human environments allowed an examination of where these two sets of factors coincide.

Risk factors for aggression and violence occur at multiple system levels, including biological (e.g., genetic, pre- and postnatal nutrition), familial (e.g., structure, income, parenting style), and personality-level risk factors. Social psychologists (like us) also investigate group-level effects (e.g., intergroup conflict), and our background readings in history, sociology, and political psychology also make it relatively easy to include factors that are normally discussed by sociologists and other social science fields (see Pettigrew, 2021, for an excellent review of this multilevel perspective – which he calls "contextual social psychology" – in prejudice, racism, and conflict).

The basic question addressed in Figure 1 is this: In what ways does rapid global warming increase the frequency of and/or intensity to which human populations are exposed to known violence risk factors at any level of science? Further, are there ways in which global warming might increase exposure to violence risk factors or decrease exposure to known protective factors (and vice versa)?

We posited that this would happen both on an individual level (e.g., many individuals will be exposed to more "risk factors" that are likely to make them violent and aggressive) as well as on a group level (e.g., conflicts over water, land). Both types will be exacerbated as the climate continues to rapidly warm and to become more unstable.

Downloaded from https://www.cambridge.org/core. Iowa State University Library, on 07 Feb 2022 at 18:20:25, subject to the Cambridge Core terms of use, available at https://www.cambridge.org/core/terms. https://doi.org/10.1017/9781108953078

The direct path that leads to increased violence and aggression is easy to observe and describe: the "direct" effect of increased heat stress on violent and aggressive behavior. This path is further examined later, but for now, simply note that there is excellent psychological and sociological evidence that when people are exposed to increased heat, they act more aggressively and violently.

The indirect paths manifest in two very different areas: individual human development (conception to adulthood) and intergroup interactions (small groups to nations). We call these pathways "indirect" because climate change itself is directly causing environmental events that *then* affect the growing individual and groups, rather than by having a direct causal impact on their physiology and psychology.

Developmentally, the changing climate can drastically alter aspects of human environments that are integral to healthy development. For example, increased droughts and famine increase chronic dehydration and malnourishment, both prenatally and postnatally, both risk factors for later aggression and violence in adolescence/adulthood. Weather disasters often pollute water and food supplies, making them less safe and potentially harmful to consume, and can also destroy communities and cause families to be broken up due to migration and forced relocation. Unstable living environments and broken families also are known risk factors for violence; they also have effects on future social perceptions, beliefs, and actions – such as making the acceptance of violent and extremist ideologies more likely.

At the highest level, these effects of climate change will increase the likelihood of group conflict. Mass migration resulting from climate change has already manifested in adverse outcomes for populations of people who have had to relocate, such as the Syrian civil war and refugee crisis (Miles-Novelo & Anderson, 2019). Additionally, increasingly popular far-right political ideologies (especially in Europe and North America) often are focused on migrants and on how they are not wanted in "our" country. As discussed earlier, rapid climate change already has forced mass ecomigrations, and soon, hundreds of millions of people will be forced to move as their homes are destroyed or made unlivable. If such anti-immigrant rhetoric persists and grows, resulting in even more harmful attitudes and behaviors toward immigrants, the ramifications and outlook for those needing a new home are potentially very dangerous. This has been seen worldwide, where aggressive stances towards immigrants are not limited to new immigrants but extend to current minority citizens as well.

Humans are, by nature, social creatures but they are also especially protective of their ingroups. In a world where resources become increasingly scarce and the physical environment becomes increasingly unstable, lashing out at out-groups is a tempting and probable outcome, one that could come at the expense

Downloaded from https://www.cambridge.org/core. Iowa State University Library, on 07 Feb 2022 at 18:20:25, subject to the Cambridge Core terms of use, available at https://www.cambridge.org/core/terms. https://doi.org/10.1017/9781108953078

of millions of lives. Civil unrest and conflict will continue to grow as our world continues to slide into chaos, especially if there is not a strong and concentrated effort to be proactive in combating both climate change itself as well as harmful intergroup attitudes and behaviors. To combat this, a major effort must be made in the implementation of public policies designed to reduce prejudice and intergroup conflict. If we do not do so, violence such as that witnessed over the water shortages in India offers a harrowing warning sign for what is to come.

Although much of this Element explores the impact of climate change on human risk factors for violence, it delves more deeply than does past psychological research on other global warming influences on humans. For instance, heat stress doesn't only increase violent thoughts, emotions, and behaviors, it also has powerful effects on brain physiology. Developmental impacts of climate change are also broader than just increasing risk factors for violence, as they will affect overall mental health, well-being, intelligence, reproduction, and almost every other human behavior we can imagine. Group-level impacts can be expanded as well, as things such as housing crises and increased participation and sympathy for extremist organizations will continue to escalate.

3 The Direct Effects of Heat On Cognition and Behavior: Route 1

Human brains and bodies are incredibly sensitive to the environment. This is obvious. But as scientists learn about the human brain, the more we discover about how the environment can greatly alter brain development and also in how our environments change our behavior. This section describes a number of ways that heat itself is a remarkable brain-changing agent. It can make us more likely to become aggressive, become more reactive and less thoughtful in ongoing social interactions, can reduce cognitive capacities, increase stress, and even cause severe brain damage.

We suspect that these sorts of direct effect might seem minuscule relative to the overall threat of climate change. However, recall our previous elaboration on risk factors on human behavior, and think about how these effects will play a role in larger scale stressors, such as mass migration. These direct effects on our brains and behaviors certainly increase the risk factors for several adverse outcomes, and when we talk about some of the larger scale impacts of climate change on societies worldwide, the direct heat effects on individuals add to the severity of some of the more "indirect" societal impacts. Additionally, there is little one can do as mitigation of some of these direct effects, as the climate is simply going to become hotter in most (and maybe all) parts of the world. Earth will have hotter summers, more

Downloaded from https://www.cambridge.org/core. Iowa State University Library, on 07 Feb 2022 at 18:20:25, subject to the Cambridge Core terms of use, available at https://www.cambridge.org/core/terms. https://doi.org/10.1017/9781108953078

people will experience heat exhaustion and stress, and there will be days that are simply too hot for our bodies to adapt to. This will happen and is happening now, so it is important to start with an understanding of how heat affects the brain and body.

The Effects of Heat On the Brain and Brain Function

Extreme heat effects on the brain carry severe consequences. It starts simply as our bodies beginning to feel tired and sluggish. As the body heats up, the brain recognizes this homeostatic imbalance and seeks to cool us down. One way of doing so is by literally forcing our body to slow down and run at a suboptimal capacity, especially if it detects that you are running low on survival resources (water, calories, etc.). Our brain seeks to find a way to achieve a state that is comfortable and in which it isn't consuming many of these resources that it may perceive as running low on or not readily available. This is your body telling you to "slow down," to find a more comfortable environment, and to replenish the resources it needs for survival.

This, of course, is coupled with other physiological changes such as perspiring, rapid heart rate, and increased oxygen flow (Kovats et al., 2008). By doing these things, the body (and brain) will cool down and be able to combat the overheating that is so dangerous to the brain. But perspiration and slowing down physical activities are often insufficient. If the body cannot adequately cool down, heat exhaustion begins to occur. The body continues to lose proper motor function, the heart continues to try to feed blood to the sweat glands, and one's respiration rate increases in part to help the heart pump blood more rapidly. For these reasons, physicians suggest sitting or lying down and to take deep breaths when one is beginning to feel heat exhaustion. One recent study (Massen, Dusch, Eldakar, & Gallup, 2014) showed that yawning could help people cope with overheating, as it forces the body to slow down and intake oxygen more efficiently, as well as slowing our heart rate. If the body cannot be properly cooled, it then begins a cycle that sends the brain and body into a frenzy (Kovats & Hajat, 2008).

What is happening neurologically is mostly determined by the hypothalamus (Boulant, 1981), which is the primary brain structure involved in thermoregulation. This part of the brain triggers the above-mentioned mechanisms used to cool off (or to heat up when too cold – such as shivering). While this is occurring, the brain is diverting resources from other areas of the brain and body to try to cool itself as quickly as possible. One side effect is that other parts of the brain are not running at full capacity. Motor functions deteriorate (triggering feeling tired and sluggish), effortful cognitive, emotional, and

Downloaded from https://www.cambridge.org/core. Iowa State University Library, on 07 Feb 2022 at 18:20:25, subject to the Cambridge Core terms of use, available at https://www.cambridge.org/core/terms. https://doi.org/10.1017/9781108953078

decision-making processes become less efficient, and there is a severe weakening of impulse control (among other effects).

The psychological effects of heat stress include impaired attention span, poorer memory, and weakened ability to process new information (Walter & Carrarreto, 2016). Basically, the ability to perceive available situational information deteriorates, which can lead to more impulsive decision-making (Vrij, van der Steen, & Koppelaar, 1994). As you might guess, impulsive/reactive decision processes are strongly linked to heightened aggression and violence (Anderson & Bushman, 2002).

Wittbrodt et al. (2018) recently reported work that examined multiple effects of extreme heat stress and lack of water availability for rehydration. Visuomotor skills (skills that require vision and movement) were severely impaired by being overheated and dehydrated. They also found evidence suggesting that brain structures are actually changing when one is dehydrated.

Heat stress also leads to other impaired functioning. For example, Chang, Bernard, and Logan (2017) showed that inducing heat stress caused individuals to perceive risk-taking behaviors as less risky in a work environment. As the authors note, although we have a good conceptualization of how the brain reacts to being too hot, and we only know of some of the cognitive indicators that it is not operating properly. Thus, we need more research to fully understand the various cognitive impairments induced by heat stress.

But what happens when we suffer heat exhaustion at an extreme level, such as heat strokes? The answer to this is simply "not good." One common effect of heat stress and exhaustion is fainting. Although fainting itself is not particularly harmful (if one falls safely and is quickly tended to), there are other extremely destructive outcomes of an overheated brain.

One effect of heat stroke is that the blood–brain barrier becomes compromised (Yamaguchi et al., 2019). When that barrier breaks down, it allows a variety of substances into brain areas in which they shouldn't be found. This can cause inflammation and other potentially serious brain damage.

Strokes, in general, can be very dangerous for our brain's health and functioning, and heat strokes offer a variety of compounding variables that can increase the damage and severity that they cause. The typical rule of thumb is that having multiple episodes increases the odds of severe damage, as does prolonged exposure to the heat while suffering from a stroke. However, even a single event of a heat stroke or heat exhaustion can cause irreparable physical damage to our brain, such as eroding the cerebellum (Walter & Carraretto, 2016).

Heat strokes have been linked to many harmful effects, inducing short- and long-term comas, personality changes, seizures, and even death (Dematte et al.,

Downloaded from https://www.cambridge.org/core. Iowa State University Library, on 07 Feb 2022 at 18:20:25, subject to the Cambridge Core terms of use, available at https://www.cambridge.org/core/terms. https://doi.org/10.1017/9781108953078

1995; Walter & Carraretto, 2016; Yaqub, 1987). Common long-term neurological impacts include cerebellar dysfunction (Deleu et al., 2005), as well as damage to brain structures such as the cerebral cortex, the brain stem, the spinal cord, and the peripheral nervous system (see Walter & Carraretto, 2016 for a full review on neurological impacts of heat strokes). Again, this leads to a variety of potentially permanent changes in behavior, including loss of motor functions, altered visio-perception, or even permanent vegetative states.

There is still much work to be done when looking at the heat's potentially devastating effects on our brains. Although the human body does much to protect itself, excessive heat opens the doorways for potential vulnerabilities to our most sensitive organ, and the impacts range anywhere from fairly minimal, to death. We now shift our attention to one of the most understood behavioral impacts of heat on humans: its ability to increase aggression and violence.

The Heat–Aggression Hypothesis

Have you ever been "hangry" – that feeling when you are so hungry that you are grouchy, touchy, or reactive? You may snap at a family member when you don't mean to, just because you're more short-tempered when you're hungry, or maybe you become more easily frustrated dealing with other issues, and you decide that before you move on, you first must deal with your hunger.

This is a common experience, one that psychologists call "irritability." The basic observation is that when humans become "irritable" (e.g., we are being deprived of some sort of equilibrium), they tend to act more impulsively, and, subsequently, more aggressively. A lot of the time, this will manifest in small and relatively nonharmful ways (like being a little short-tempered when you're hungry), but other times this can be the physiological underpinning to more severe and harmful behaviors (Miles-Novelo & Anderson, 2019). Irritability alone doesn't totally explain violent or aggressive behavior, one must have several other risk factors at play before that is the case, but it can be a very powerful yet subtle "push" over the edge. One of the most common effects we see in this research literature comes from a very basic finding: people tend to become more aggressive as they get "hotter."

Again, this effect is rather subtle, but it seems intuitive. Common metaphoric language describes people who are frustrated and angry as "hot," "steaming," or "hot under the collar." Typically, impulsive and risky behaviors are associated with warm colors and words. There seems to be a direct way in which heat makes us act more aggressively, but it doesn't stop there. When we are irritated, we also *perceive* the world around us, including the way others are behaving, as

Downloaded from https://www.cambridge.org/core. Iowa State University Library, on 07 Feb 2022 at 18:20:25, subject to the Cambridge Core terms of use, available at https://www.cambridge.org/core/terms. https://doi.org/10.1017/9781108953078

being much more aggressive. So, heat stress not only primes people to act more aggressively, but hot people also are much more likely to perceive others as behaving aggressively, which further increases the odds that hot people will respond to others in a hostile manner (Anderson & Bushman, 2002; Berkowitz, 1989; Miller, Pedersen, Earleywine, & Pollock, 2003; Zillmann, 1978). When this happens, we become much more likely to respond aggressively to our perceived provocateurs, starting a cycle of aggressive conflict (Anderson, Buckley, & Carnagey, 2008).

Although our analogy of being "hangry" is not perfect in describing the "direct effect" of heat on humans, heat stress's causal effect on aggressive affect, cognition, and behavior has been known for some time (for reviews, see Anderson, 1989, 2001; Anderson & Anderson, 1998; Anderson, Anderson, Dorr, DeNeve, & Flanagan, 2000). There are three main methods used to examine data on this relationship between heat and aggression: 1) through brief experimental studies, in which participants are randomly assigned to hot or cold conditions, 2) through geographic region studies, in which rates of violence are compared between geographic areas with different climates, and 3) through time-period studies, in which rates of violence and crime are compared across different time periods that differ in temperature (Plante, Allen, & Anderson, 2017). All three methods have arrived at the same conclusion: hotter temperatures are a significant, stable, and independent predictor of violence. Examining each method allows an understanding of how they build on one another, with experimental studies explaining the effect on an individual level and geographic and time-period studies showing how this can manifest on a larger scale. Let us start by looking at the experimental evidence, then building on that with the geographic and time-period studies.

Experimental Studies

First, note that creating valid experimental designs to study aggression and violence is inherently complicated. This is something that has been noted in much of our research team's media violence work, where we must design creative paradigms that mimic aggression and violence without allowing participants to seriously harm one another. However, researchers have carefully constructed ways to measure these constructs in the laboratory, and the results from these studies have an incredible impact because experimental studies (sometimes called randomized control studies) allow strong causal inference.

When a person perceives another as behaving inappropriately aggressively towards them, they are more likely to retaliate aggressively (Anderson & Bushman, 2002; Anderson et al., 2008). This basic fact makes it easy to look

Downloaded from https://www.cambridge.org/core. Iowa State University Library, on 07 Feb 2022 at 18:20:25, subject to the Cambridge Core terms of use, available at https://www.cambridge.org/core/terms. https://doi.org/10.1017/9781108953078

at how heat impacts one's perceptions of another person. If heat stress increases such aggressive perceptions, thoughts, and/or feelings, it is likely that heat stress will also increase aggressive behavior. Multiple studies have shown such effects. One study had participants view four video clips of adult couples having conversations. After each clip, participants rated how aggressive or hostile they perceived those recorded conversations to be. Participants had been randomly assigned to do the video clip task in either an uncomfortably warm/hot room or in a room at a comfortable temperature (Anderson et al., 2000). Uncomfortably warm participants were not only more likely to be hostile themselves than comfortable participants (thereby replicating similar effects found in Anderson et al., 1996) but also perceived the video clip interactions as containing more hostility and aggression. This effect works not only when participants are hot themselves, but also when they are only primed with the *idea* of heat (e.g., Wilkowski et al., 2009). They found that participants who were exposed to images related to heat were more likely to judge neutral facial expressions as aggressive and were more likely to have aggressive thoughts than control condition participants.

Early experiments of heat effects on actual aggressive behavior were plagued by methodological problems and yielded mixed results (Anderson et al., 2000). However, that same review article reported a series of new experiments that clearly demonstrated heat stress effects on immediate retaliatory aggression.

Another interesting experiment examined the effects of heat stress in a police firearms training program (Vrij, van der Steen, & Koppelaar, 1994). This system presented police officer participants with a video-based burglary scenario in which a person suddenly appears with a crowbar in hand. Participants had to quickly decide whether to draw their weapon (a laser gun) and, if drawn, whether to shoot the suspect. Those who had been randomly assigned to complete this scenario in a hot room reported more aggressive and threatening impressions of the suspect and were more likely to draw and fire their weapon than those assigned to complete the training in a room set to a comfortable temperature.

Although this latter study isn't a clear example of actual aggression (i.e., behavior intended to harm a real person who wishes to avoid it), overall, these and other experimental studies indicate that heat stress can cause increased aggression-related judgments, perceptions, thoughts, feelings, and aggressive behaviors at the individual level. The evidence from studies like these shows that as people get hotter, they become impulsive, more likely to perceive others as aggressive and hostile, and are more likely to behave aggressively themselves.

Downloaded from https://www.cambridge.org/core. Iowa State University Library, on 07 Feb 2022 at 18:20:25, subject to the Cambridge Core terms of use, available at https://www.cambridge.org/core/terms. https://doi.org/10.1017/9781108953078

Overall, the experimental data paints a clear causal picture of how the direct heat effect works on individuals. This allows consideration of group-level data with a good understanding of how individual-level mechanisms can explain group-level heat effects.

Comparing Geographic Regions

One type of group-level data involves comparing rates of violent crime across geographical regions that have different climates. For example, Mares and Moffett (2015) analyzed violent behavior in sixty different countries from across the globe and found a positive link between heat and violence. Each Celsius degree increase in temperature was associated with a 6 percent increase in the homicide rate. This effect was magnified in countries that were also mired in some sort of conflict and/or instability.

Ideally, geographic region studies are done across regions that are similar in other key violence risk factors or with datasets that allow such alternative risk factors to be statistically controlled. This allows tests of the plausibility of many alternative explanations (e.g., poverty, culture).

Such similar-region and statically controlled studies have been done; they consistently find that hotter regions (such as cities across the United States) have higher rates of violent crime, even when controlling for as many as fourteen other risk factors (e.g., poverty, age distribution, racial composition, Southern honor culture, and unemployment; Anderson, 1989; Anderson & Anderson, 1996). Interestingly, some of these geographical region studies have also assessed nonviolent crime rates; they generally find weaker or no heat effect on nonviolent crimes, as expected by the heat hypothesis.

An additional interesting finding is that, although nontemperature risk factors have their own independent effects on violent crime, some data suggests that such nontemperature risk factors can “amplify” the heat effect on violent crime (Harries & Stadler, 1983; Van de Vliert, 2009).

Of course, there are significant interpretation issues in comparing geographic region studies. Although researchers have controlled for many potential risk factors that could potentially operate as confounds in detecting this effect (socioeconomic status (SES), poverty, etc.), such correlational studies always leave room for doubt about causality; perhaps there are other unmeasured differences between the regions.

The next type of group data allows tests of the heat hypothesis that are free of region-based alternative explanations. Specifically, behavioral scientists have examined the heat hypothesis within the same region but over different periods of time that differ in temperature.

Downloaded from https://www.cambridge.org/core. Iowa State University Library, on 07 Feb 2022 at 18:20:25, subject to the Cambridge Core terms of use, available at https://www.cambridge.org/core/terms. https://doi.org/10.1017/9781108953078

Comparing Violence Over Time

Numerous studies have used various time periods, including three-hour time blocks, days, months, seasons, even years to look at differences in violence in a specific geographic area. The logic of such studies is that one can assume that the cultural and demographic variables in a given location remain largely unchanged, barring any kind of extreme event or shift (e.g., large seasonal shifts in population). For areas that remain stable over the time period assessed, examining the association between temperature and violent crime rates over time provides a third way to test the heat hypothesis.

In studies such as these, the picture is clear: hotter periods of time yield higher rates of violence (for reviews, see Anderson, 1989, 2001; Anderson & Anderson, 1998; Anderson et al., 2000; Anderson & Delisi, 2011). These results are stable and are found using various time blocks, including years, seasons, days, or even hours (Anderson, Bushman, & Groom, 1997; Bushman et al., 2005a, 2005b). For example, Anderson et al. (2000) reported that there are about 2.6 percent more assaults and murders in the United States during the summer than in other seasons of the year. They also found that hotter summers yield a bigger increase in violence than cooler summers. Furthermore, violence rates are higher in hotter years than in cooler years, even after key statistical controls are applied.

Other time-period studies provide additional and consistent results. Aggression rates – as measures by murder, rape, and assault rates (e.g., Anderson & Anderson, 1984), spontaneous riots (Carlsmith & Anderson, 1979), domestic violence (e.g., Auliciems & DiBartolo, 1995; Rotton & Frey, 1985), assaults on bus drivers (Yasayko, 2010), aggressive honking at other drivers (Kenrick & MacFarlane, 1986), and even the rate of batters being hit by pitched baseballs (Reifman et al., 1991) – are higher during hotter days, months, seasons, and years. Such findings have occurred with data from cities as varied in climate as Brisbane, Australia, Vancouver, Canada, and the US cities of Chicago, Minneapolis, Dallas, and Houston.

Additional analyses on the US city data give a more concrete idea of the magnitude of heat on annual "serious & deadly assault" rates (Anderson & DeLisi, 2011). A 1.1 C° increase in annual temperature is associated with up to 25,000 more cases of serious and deadly assault per year in the United States.

Mechanisms Underlying Direct Temperature–Aggression Effects

So, what exactly are the mechanisms behind why heat causes increased violence and aggression? There are a handful of theoretical rationales behind the link between heat and aggression, and often scholars automatically pit these theories

Downloaded from https://www.cambridge.org/core. Iowa State University Library, on 07 Feb 2022 at 18:20:25, subject to the Cambridge Core terms of use, available at https://www.cambridge.org/core/terms. https://doi.org/10.1017/9781108953078

against one another. However, we challenge this automatic tendency and demonstrate how many of these theories are compatible. Additionally, when synergized, these theories help create a holistic understanding of the relationship between heat and aggression, as different theories help explain different aspects of the relationship in different contexts.

First, consider strictly *physiological* mechanisms. As described earlier, we know that the brain is responsible for thermoregulation and has a variety of mechanisms designed to keep us cool and functioning. However, the part of the brain that is responsible for thermoregulation is the same part of the brain that is responsible for emotion regulation (Anderson, 1989; Boyanowsky, 1999, 2008; Boyanowsky et al., 1981). As noted before, the brain has finite resources that can be devoted to functioning. When the part of the brain needed to regulate emotion is already activated in trying to cool ourselves down physiologically, we must either offload those resources elsewhere in the brain or suffer performance issues. Additionally, the body has other reactions to the heat, such as producing more adrenaline (Simister & Cooper, 2005), which can lead to more reactive and therefore aggressive behavior in certain conditions (such as when provoked). This research demonstrates what we have called the "hardwired" aspect of heat's impact on aggression (Anderson 1989; Miles-Novelo & Anderson, 2019).

Now, consider some basic *psychological* findings that also underpin the heat/aggression relation. The first comes from embodied cognition research, which suggests that humans are incredibly responsive and susceptible to the stimuli in their environment, which ultimately influences how we think and feel (Wilkowski et al., 2009). Research shows that hot temperatures produce discomfort and irritability, and then we can clearly see how being in an uncomfortably hot environment can yield biases in aggression-related precursors such as hostile perceptions, hostile feelings, and aggressive inclinations.

At first, there may be confusion as to how the basic physiological functions interact with these psychological findings. However, we contend that these findings are, in fact, fully compatible. Although there may be an independent physiological effect, more social psychological factors (irritability, biases in social perceptions) can be a direct consequence of these physiological effects of heat on emotion regulation. There are some effects that we often lump together as "irritability" (see Miles-Novelo & Anderson, 2019), as there may not be a way (currently) to piece apart the physiological impacts from the psychological ones. That is, heat stress may simultaneously cause both physiological and psychological effects that increase the likelihood of an aggressive (or violent) act emerging.

Downloaded from https://www.cambridge.org/core. Iowa State University Library, on 07 Feb 2022 at 18:20:25, subject to the Cambridge Core terms of use, available at https://www.cambridge.org/core/terms. https://doi.org/10.1017/9781108953078

Another important theory to consider at this juncture is called "routine activity theory" (RAT; Cohen & Felson, 1979). Routine activity theory simply states that as our environment changes (e.g., church, school, bar, time of day, day of week), so does the repertoire of likely thoughts, feelings, and behaviors. Thus, some portions of observed differences in aggression during hot/cool weather might be caused by changes in routine activities. That is, when it's warmer outside (but not exceptionally hot), people tend to engage with more people outside and engage in behaviors that are more social, increasing the opportunity for aggression towards others. For example, during a heatwave, those lacking access to air conditioning may spend more time outside or may go to an air-conditioned bar and drink more beer. Being outside increases encounters with others, and we all know too well the impact alcohol can have on aggressive behaviors and cognitions. This is just one specific example, but a good one to show how the weather impacts our behaviors and how we structure social interactions around it. RAT has quite a lot of data that suggests that it holds merit, such as studies showing that weekends tend to yield higher rates of violent crime than weekdays (Anderson & Anderson, 1984; Anderson et al., 2000). However, many of the studies reviewed earlier show that RAT cannot fully explain the heat–aggression findings. Instead, it is but one piece of the puzzle.

There also are culture-based theories of aggression, such as the distinction between honor and dignity cultures (e.g., Uskul & Cross, 2020). For example, Cohen and Nisbett's (1994 theory about the development of the Southern culture of honor in the U.S. has been used to claim that the heat/aggression effect in U.S. cities is totally spurious because Southern cities have both relatively high temperatures and residents who have adopted a relatively violent culture. Subsequent studies that directly tested this potentially confounding hypothesis have still yielded clear support for the heat hypothesis independent of such cultural variables (Anderson & Anderson, 1996). To reiterate, we view cultural effects on violence rates as a real phenomenon that works in tandem with the heat hypothesis.

A recent theoretical model, known as CLASH (Climate, Aggression, and Self-Control in Humans), seeks to rectify some of these issues. CLASH posits those cooler climates put more of a cultural emphasis on the future rather than the present, more value in self-control, and have a "slower life history strategy" than cultures in warmer climates (Van Lange, Rinderu, & Bushman, 2017). Although more research is needed to test the CLASH model, note that both CLASH and RAT can help explain that although the direct heat/aggression effect is real, certain moderators can mitigate or exaggerate this effect, whether

Downloaded from https://www.cambridge.org/core. Iowa State University Library, on 07 Feb 2022 at 18:20:25, subject to the Cambridge Core terms of use, available at https://www.cambridge.org/core/terms. https://doi.org/10.1017/9781108953078

that be by changing patterns of behaviors and environments, or because of cultural norms.

But Some Data Shows that Crime Rates Are Decreasing?

This is a very important question that comes up when talking about the research mentioned, and in general, when thinking about how rapid climate change is going to impact violence and aggression across the globe. We (the authors) don't doubt the observable data that, in general, crime statistics in some countries are going down. In fact, a recent article published by the Pew Research Center conglomerated crime data from both the United States FBI and Bureau of Justice Statistics (BJS) and noted that the BJS reported that violent crime rates had fallen somewhere around 74 percent from 1993–2019 (Gramlich, 2020). Similarly, the FBI has reported drops of around 68 percent in the number of robberies, 47 percent in murder/nonmanslaughter cases, and 43 percent in aggravated assaults in that same timeframe.

However, this does not invalidate or contradict the general hypothesis that heat increases aggression and violence. In fact, much of the evidence collected has found that increases in violent crime still occur over hotter periods of time or in hotter geographic regions, *independent* of the fact that overall crime statistics are decreasing. What we see is that even though overall crime has reduced over time, there are predictably more reports of crime (notably, violent crime) when the temperature is hotter. The same is true when looking at reports of aggression and violence across varying geographic regions, with hotter regions still reporting higher rates of crime. However, more research needs to be done to model how the relationship between heat and crime exists as we continue to see overall decreased rates of crime.

Additionally, as mentioned before, predicting violence and aggression means considering numerous risk factors, of which heat is only one. Simple crime rate statistics that don't consider other key risk factors that change over time are largely irrelevant. For example, during the same time period in which violent crime rates have declined, there have been massive changes in the population's age distribution (e.g., declines in high-risk age demographics and increases in low-risk ages), declines in childhood lead exposure by high-risk ages, and massive diminution of the drug wars.

Consider our model of how rapid global warming can increase aggression and violence. Many of the global warming-related risk factors are *indirect*; the consequences haven't fully begun to emerge. Some of them will take eighteen years or so to emerge (i.e., those that create violence-prone adults). Resource scarcity, mass migration, droughts, famines, more severe and frequent natural

Downloaded from https://www.cambridge.org/core. Iowa State University Library, on 07 Feb 2022 at 18:20:25, subject to the Cambridge Core terms of use, available at https://www.cambridge.org/core/terms. https://doi.org/10.1017/9781108953078

disasters, will all have global-level impacts that increase the risk factors people are exposed to. Although it is clear that heat itself has a direct impact on our behavior, much of the direct effect can be mitigated by reducing these other risk factors (access to resources, political and governmental stability, inclusive environments, income distribution, etc.). However, the much more impactful and frightening result of climate change will be how it undermines these other factors, making it much more likely for violence and conflict to occur.

However, these direct heat effects and their underlying mechanics are not the only paths by which rapid global warming will increase aggression and violence. Additionally, this pathway is perhaps the most "uninteresting," offers the least amount in terms of potential interventions, and is the least preventable. Rather, the focus of society's energy and attention should be directed at the more subtle impacts climate change will have on violence, aggression, and conflict. These two indirect pathways offer the most opportunity for policy and social intervention. There is little we can do about the direct heat effects (other than changing behaviors worldwide to curb greenhouse gases). However, there is much we humans can do to short-circuit the operation of these indirect effects (Routes 2 and 3). These indirect effects are discussed in our next two major sections.

4 Indirect Effects of Climate Change On Aggression and Violence

This section examines more broadly how rapid climate change influences many aspects of life and how some of those changes will increase human aggression and violence. Recall from our discussion of risk factors that no one variable or event causes people to behave or think in a specific way. Rather, there are many combinations of environmental, genetic, biological, sociological/cultural, and psychological factors that make any given behavior or thought more or less likely.

Climate change is rapidly changing the frequency and prevalence of many risk factors known to contribute to violent behavior. In this section, we highlight many of them, from factors that operate on individual levels (e.g., developmental factors such as malnourishment) as well as on group and societal levels (e.g., unstable governments and economies). By examining how climate change is expected to exacerbate these risk factors, we can better understand how our changing planet will also change humans in general and can inspire science-based interventions designed to reduce the harmful effects.

These indirect effects mostly manifest as the result of things such as the impact of natural disasters (that are becoming more common and severe), mass migration, political instability, and adverse socioeconomic outcomes. For

Downloaded from https://www.cambridge.org/core. Iowa State University Library, on 07 Feb 2022 at 18:20:25, subject to the Cambridge Core terms of use, available at https://www.cambridge.org/core/terms. https://doi.org/10.1017/9781108953078

instance, we know that growing up in an unstable living situation is a huge predictor of several adverse life outcomes, such as negative impacts on social-emotional development, cognition, and behavior (Sandstrom & Huerta, 2013). It also increases the likelihood of later violent criminal behavior. We also know that climate change is going to increase situational factors that lead to more unstable living circumstances (increased droughts, resource scarcity, civil conflict) for more families, thus impacting more developing children every year. These changes to life circumstances, if not properly addressed and planned for, can cause very stressful (or even traumatic) conditions for children to grow up in, which will harm their development into adulthood. This is just one of the very many examples we will explore in this section.

Route 2: Creating Violence-Prone Adults

Much of what we've already discussed about the direct effect of heat on cognition happens at the "individual" level. That is, being exposed to heat is something that impacts each individual human being in a specific way. With that direct effect in mind, we now explore how climate change, albeit via indirect means, will influence behavior on an individual level. We are going to look at this through two main lenses, climate change's impact on important human developmental factors, as well as how it increases other sociological/political risk factors for aggressive and violent behavior.

Increased Prevalence of General Developmental Risk Factors

Human development is one of the most studied areas of psychology. The ambient physical, social, and cultural environments are integral to the human development process, and we know that climate change is already drastically altering those environments. It's easy to see the devastating changes that are occurring because of climate change; floods caused by increasing sea levels and more intense storms, increased droughts that lead to devastating wildfires, more volatile and severe natural disasters, increased civil conflicts because of economic and political instability are all caused by the rapidly changing planet (Miles-Novelo & Anderson, 2019). These sorts of event are drastic enough to potentially harm the surrounding environments that can be vital for human development. People are greatly affected by their living situations, where more positive and loving environments often lead to more positive developmental outcomes than seen in comparatively negative environments. In this section, we briefly summarize some of these negative environmental risk factors and how they become more common and more severe as a result of rapid global warming.

Downloaded from https://www.cambridge.org/core. Iowa State University Library, on 07 Feb 2022 at 18:20:25, subject to the Cambridge Core terms of use, available at https://www.cambridge.org/core/terms. https://doi.org/10.1017/9781108953078

When thinking about risk factors, note that they do not occur randomly across populations. Rather, human developmental risk factors tend to be comorbid; some people (from conception through adulthood) tend to have many risk factors, while others have few (Sandstrom & Huerta, 2013). Additionally, it is through factors such as family income and socioeconomic status that many adverse developmental outcomes operate. In their excellent review of developmental factors affected by instability, Sandstrom and Huerta (2013) refer to this as a “domino effect” and point out how low-income or even middle-income families can struggle to have saving assets to handle transitional periods. One can easily imagine the many ways in which climate change could negatively influence a family with low resources, such as making emergency transitions a much more frequent occurrence for middle- and low-income families around the world.

Here, we focus on how some of these changes and instabilities influence individual development. Note that we don’t claim universality of what defines “normal” or “stable”; different families in different places around the world have different cultural expectations and norms. The risk factors that we discuss are those that are somewhat generalizable across differing cultures (for a more comprehensive understanding of how cultural differences in development could be affected by climate change, see Kuwabara & Smith, 2012; Ogbu, 1988).

One particularly strong impact on individual development is family stability. This can manifest in a variety of forms, and for many families, multiple stability-decreasing factors occur simultaneously. This can include economic stressors (low or inadequate family income), unstable housing situations, parental job instability, and lack of parental presence. About one in three children in the United States experiences major family structure change by age six, whether that was from parental death, divorce, separation, or some form of new cohabitation (such as a new adult becoming present in their living situation; Pew Research Center, 2015). Some projections forecast that the percentage of children growing up in single-parent homes, at least temporarily, could become as high as 50 percent globally, in part, due to the environmental challenges presented by rapid global warming (Sandstrom & Huerta, 2013). We already see a pattern more broadly in immigration, especially when relocation is caused by sudden devastating events, such natural disasters. These kinds of events often serve as a basis for the displacement of families and often also can result in the permanent fragmentation of them (Rabin, 2018). This can be because of direct immigration policies or because of the causal event itself, whether it be war, famine, or a natural disaster. Because of the direct consequences of rapid climate change on our environment, we know that billions of people are at risk of displacement, and therefore, at higher risk of family separation.

Downloaded from https://www.cambridge.org/core. Iowa State University Library, on 07 Feb 2022 at 18:20:25, subject to the Cambridge Core terms of use, available at https://www.cambridge.org/core/terms. https://doi.org/10.1017/9781108953078

Although these changes are not always negative (divorce or separation could reduce parental conflict), Sandstrom and Huerta (2013) do note that most experts consider a change in parental dynamics or family structure as a cause of severe anxiety and stress, which can lead to emotional instability in both the caregivers and the children.

Growing up in a home where parents who were living together when the child was born but are separated by the age of five leads to poorer vocabulary skills for that child, poorer prereading skills, and increased aggressive behaviors (Craigie et al., 2012). Similarly, rates of obesity and asthma are higher in these circumstances (Craigie et al., 2012; Sandstrom & Huerta, 2013). In addition to its well-known effects on heart disease, arthritis, and other medical conditions, obesity has been found to negatively affect brain plasticity, making it harder for people to learn and retain how to do new tasks (Sui et al., 2020). Moving from a single-parent home into an unmarried, unstable cohabitation with a new partner can also lead to decreased academic performance and engagement (Sandstrom & Huerta, 2013). Such family instability predicts several adverse long-term outcomes, such as failure to complete high school (Sandstrom & Huerta, 2013). In a world that is increasingly plagued by the impacts of rapid climate change (e.g., natural disasters, mass migration, war), impacts that are known to be devastating to healthy family structures and stability, it seems obvious that the impact on developing children will be especially harmful.

Naturally, this extends to economic and housing stability. Research finds that the parents having unstable employment is associated with adverse academic outcomes, such as more frequent grade repetition (grade retention), lower educational attainment, and internalizing and externalizing behaviors (Sandstrom & Huerta, 2013). Children whose parents have frequent involuntary job instability often have worse health outcomes, even more so than children whose parents have voluntary job fluidity, work a stable but low-wage job or have inconsistent work hours. Often these effects are not necessarily a reflection on the parent or parents themselves, but are, rather, a product of the situation. When families face such major life stressors, oftentimes the parents must devote more time, energy, and resources to addressing external concerns rather than being able to adequately handle important internal family responsibilities. Therefore, much of the developmental literature has for decades strongly urged societies to strengthen social safety nets and improve access to monetary resources for struggling families. It should come as no surprise that such actions are needed in all nations to combat the impacts of climate change (which are discussed in the last section of this Element).

Downloaded from https://www.cambridge.org/core. Iowa State University Library, on 07 Feb 2022 at 18:20:25, subject to the Cambridge Core terms of use, available at https://www.cambridge.org/core/terms. https://doi.org/10.1017/9781108953078

For families who experience this instability across countries, these disadvantages are even more pronounced. When a family immigrates or migrates, the effects on children (both those who moved and those who were born after the relocation but before successful integration) are potentially very harmful, especially if their new permanent location lacks support for the families that arrive there. Coll et al. (2012) notes that these children must learn to adapt and grow in not only a potentially completely different environment than where they are from (or that they experience at home) but often that adjustment comes with little guidance from parents and other older family members, as they presumably are adjusting to their new environments as well. Parents struggling to learn a new language, for instance, often have difficulty finding and keeping adequate jobs, engendering economic and income instability. Additionally, migration often impairs language acquisition in children. We know that language acquisition is easier for children who are younger than it is for adolescents, young, and older adults. Thus, acquiring the new country's dominant language is differentially difficult depending on the child's age and on the parents' facility for the new language (Coll et al., 2012).

Migrating families also must deal with being seen as a potential "outgroup" in their new community and oftentimes face additional stress and anxiety due to perceived and actual stereotyping, prejudice, discrimination, rejection, and lack of community support. Later, we discuss more how climate change increases negative perceptions of "outgroups" (and, subsequently, increased tensions along the lines of race, ethnicity, religion, and socioeconomic class), but for now, just note that having these experiences leads to a host of long-term adverse outcomes for children and adolescents.

One way to think about this is the concept of "adverse childhood experiences" (ACEs; McCoy, Tibbs, DeKraai, & Hansen 2021). The impact of ACEs has been shown to be predictive of many adverse and risky behaviors, such as likeliness to engage in drug and alcohol use, increased chances of early death, and increased likelihood of aggressive and violent behavior in adolescence and adulthood. In some circumstances, having multiple ACEs increases susceptibility to extremist ideologies and results in the engagement of terrorist activities.

ACEs not only affect development at behavioral and emotional levels, they also have neurological and cognitive consequences (McCoy, Tibbs, DeKraai, & Hansen, 2021), including deficits in executive functioning, attention, abstract thinking, memory, and language (De Bellis, 2005; Eigsti & Cicchetti, 2004; Eisen, Goodman, Qin, Davis, & Crayton, 2007; Wilson, Hansen, & Li, 2011). Many of these adverse outcomes are directly linked to the likelihood of later violent criminality (e.g., Vazsonyi, Flannery, & DeLisi, 2018).

Downloaded from https://www.cambridge.org/core. Iowa State University Library, on 07 Feb 2022 at 18:20:25, subject to the Cambridge Core terms of use, available at https://www.cambridge.org/core/terms. https://doi.org/10.1017/9781108953078

Rapid climate change is already reducing access to a wide range of resources by increasingly large segments of the world's population, such as education, safe water, and proper nutrition. For example, rapid global warming is causing prolonged droughts, which leads, in turn, to water scarcity and failed crops. This means fewer necessary nutritional resources are available from the date of conception throughout development, and also that those fewer resources are mostly going to places that can afford to buy them. There are a host of developmental consequences for those who lack access to these resources. For example, cognitive performance in children can drop significantly if their diets severely lack protein (Scrimshaw, 1998). We also know iron deficiency is the most common manifestation of malnourishment, and that can impact the development of brain enzymes that are essential to the development of cognitive skills, as well as in the development of behavior, and that prenatal and infant iron deficiencies can lead to adverse development that is irreversible (Scrimshaw, 1998). We have already discussed the impacts of dehydration on the brain, but it is important to note that those effects are even more harmful and impactful on developing brains.

Rapid climate change increases the frequency of ACEs in yet another way, through pandemics. This is especially true for families with low incomes, much like how adverse outcomes (both directly in who experiences the worst sickness from the virus, as well as the downstream economic and societal level impacts) during the COVID-19 pandemic have disproportionately hurt poorer families (McClure et al., 2020). Such families worldwide are much more likely to get the disease (COVID-19 and future pandemics), because of several factors, including lack of resources and education about the disease, being much more likely to live in overcrowded communities, representing much more of the designated "essential" workforce not able to quarantine or work from home, not being able to house children and needing to still send them to school, lack of access to outdoor space, lack of access to testing and treatment, as well as disproportionately meaning that contracting the pandemic disease will be both more likely and more severe (McClure et al., 2020).

To make matters worse, scientists have noted that scenarios like the COVID-19 pandemic are going to become more common as rapid climate change continues. On the page for Harvard's T.H. Chan School of Public Health, Aaron Bernstein speaks on several of these issues, demonstrating how the COVID-19 pandemic serves as a case study for future pandemics that will be caused by climate change and how rapid climate change facilitates the increase in exposure to more harmful pathogens. One of the main mechanisms is that as the more rapidly climate heats up, the more species will migrate to cooler climates. This forces species that normally have minimal or no contact at all

Downloaded from https://www.cambridge.org/core. Iowa State University Library, on 07 Feb 2022 at 18:20:25, subject to the Cambridge Core terms of use, available at https://www.cambridge.org/core/terms. https://doi.org/10.1017/9781108953078

to suddenly be in close proximity. This creates more opportunities for diseases to spread or be curated. Additionally, as species continue to die off (both plants and animals), it will force those that survive to consume food from untraditional places, again offering a mechanism for pathogens to spread to new areas, across species, and also for new pathogens to evolve (Harvard T.H. Chan School of Public Health, 2020).

Increased Prevalence of Developmental Risk Factors For Violence-Prone Behavior

As implied in the previous section, rapid climate change exposes a developing human – from prior to conception (i.e., via epigenetic effects on the mother) through young adulthood – to causal risk factors known to increase the likelihood of that person becoming a violence-prone adult. As the climate continues to change rapidly and create global instability, a greater proportion of children will be exposed to many major risk factors if there are not societal-level interventions to mitigate the potential damage. Delineations of many aggression/violence risk factors can be found in Anderson and Carnagey (2004), Plante, Anderson, and Delisi (2017), and Vazsonyi, Flannery, and DeLisi (2018).

Basically, the major direct effects of rapid global warming on the earth's physical systems (e.g., more severe and frequent droughts, tropical storms, local storms, sea-level rise, floods, water shortages, and access to food) *indirectly* increase exposure to known risk factors that can influence the development of violence-prone individuals. These include (among others) poverty, dysfunctional parenting, disrupted families, exposure to neighborhood and community violence, exposure to war and civil conflicts, poor prenatal and childhood nutrition, poor maternal nutrition, and poor living conditions (Anderson & DeLisi, 2011; Vazsonyi, Flannery, & DeLisi, 2018).

One recent study reviewed twenty-eight extreme weather events across continents. The research team found that half of these weather events were either caused by human-induced climate change or were at least exacerbated by it (Herring et al., 2015). And, of course, each event increased exposure to risk factors for creating violence-prone people.

Some research has shown that genetic factors interact with social, environmental factors in creating violence-prone adults. One study examined a specific interaction between monoamine oxidase A (MAOA: an enzymatic degrader that moderates neurotransmitters) and childhood maltreatment in relation to later antisocial behavior and outcomes. In very simple terms, this enzyme has an impact on the production and release of amines such as dopamine and serotonin,

Downloaded from https://www.cambridge.org/core. Iowa State University Library, on 07 Feb 2022 at 18:20:25, subject to the Cambridge Core terms of use, available at https://www.cambridge.org/core/terms. https://doi.org/10.1017/9781108953078

and the researchers wanted to know whether exposure to childhood maltreatment could impact the development of this enzyme, thus impacting other brain functions and potentially causing behavioral disorders (Caspi et al., 2002). What they found (and has been replicated) was that the relationship between child maltreatment and antisocial behavior was conditional on a person's MAOA genotype. In one sample, only 12 percent had had both risk factors but accounted for 44 percent of the violent crime convictions by the total sample. Similarly, 85 percent of people who had both risk factors developed some form of antisocial behavior. But, in the absence of maltreatment, the genetic risk factor did not show up in violent criminality or broader indicators of antisocial behavior.

In this and subsequent paragraphs, we focus on four other key factors: food/water insecurity, economic deprivation, susceptibility to terrorism, and preferential ingroup treatment.

Even in the United States, before the COVID-19 pandemic, 10.5 percent of households faced food insecurity (US Department of Agriculture, 2020). Subsequently, that number increased to estimates as high as 25 percent (Schanzenbach & Pitts, 2020). Global figures for food insecurity are generally much worse. Scientists from several domains generally find that food insecurity, poor prenatal nutrition, and poor postnatal nutrition can lead to increases in aggressive and antisocial behavior in children. One classic example is a study on Mauritanian children, which looked at their nutritional intake and its relationship to behavioral disorders. What the researchers found was that malnourishment at three years old increased the likelihood of hyperactivity and aggression when they were eight years old and of behavioral conduct issues and disorder at age eleven (Liu et al., 2004). All four of these childhood behavior problems are major risk factors for antisocial and violent behavior in adulthood (Anderson & Carnagey, 2004; DeLisi, 2005; Warburton & Anderson, 2018).

Another relevant well-known finding comes from a study conducted by looking at 100,000 Dutchmen born shortly before and after World War II (Neugebauer, Hock, & Susser, 1999). What makes this cohort interesting is that, from October 1944 until May 1945, there was a German blockade that split the Netherlands. This had a major effect on food supplies to different parts of the country, as resources were held up on account of the blockade. The researchers used this historical event to create two comparison groups, those whose mothers experienced malnourishment during the first and second trimesters of pregnancy and those whose mothers had not. Men whose mothers had experienced malnourishment during fetal development were 250 percent more likely to develop antisocial personality disorder than were the well-nourished ones.

Downloaded from https://www.cambridge.org/core. Iowa State University Library, on 07 Feb 2022 at 18:20:25, subject to the Cambridge Core terms of use, available at https://www.cambridge.org/core/terms. https://doi.org/10.1017/9781108953078

This relationship between malnourishment and antisocial aggression is well established (e.g., DeLisi, 2005; Huston & Bentley, 2009; Plante, Allen, & Anderson, 2017). For example, malnourishment has a large impact on the release of cortisol in the mother during her pregnancy, leading to numerous adverse developmental outcomes in the fetus (Chen, Cohen, & Miller, 2010).

Other studies have specifically focused on climate change's effect on violence. For example, one study found that each standard deviation increase in rainfall and warmer temperatures was associated with a 4 percent increase in interpersonal violence and a 14 percent increase in intergroup violence[2] (Hsiang, Burke, & Miguel, 2013).

The Intergovernmental Panel on Climate Change has issued numerous warnings about the damage rapid climate change will have (and already is having) on agricultural production, greatly diminishing food supply (IPCC, 2007, 2013). Knowing how malnutrition affects development is key to creating effective mitigation and prevention strategies for those most at risk. Specifically, the IPCC has reported that global warming will cause a drop in crop yields, decrease the amount of grazeable land for livestock, and the outright loss of farmable land because of droughts, wildfires, increased humidity, and flooding (IPCC, 2007, 2013).

The effects of poverty and malnutrition on human development are inherently linked; thus, it is also important to understand related poverty-linked violence risk factors. Poverty leads to increased exposure to numerous risk factors, such as decreased life satisfaction, increased resentment, and dissent (Doherty & Clayton, 2011). Not only does material inequality increase these risk factors, but so does the *perception* of inequality. Perceived inequality of any kind (race, gender, class, etc.) has been found to be a potential motivation for enacting violent revenge at both the individual level (Route 2) and group level (Route 3) (Archibald & Richards, 2002; Cramer, 2003; Hage, 2003). This effect is exacerbated when the perception of inequality increases rapidly and when that perception leads to anxiety about one's future (Goodhand, 2003; Nafziger & Auvinen, 2002). It is important to note, however, that poverty itself does not create violence. The conditions often surrounding impoverished communities and families (that is, the systems that create poverty and that encourage inequality) are what create violent outrage (Barnett & Adger, 2007). This discussion leads to the third route through which rapid global warming increases violence.

---

[2] Intergroup violence will be discussed more thoroughly in the next section, on Route 3 linking climate change to violence, in Figure 1.

Downloaded from https://www.cambridge.org/core. Iowa State University Library, on 07 Feb 2022 at 18:20:25, subject to the Cambridge Core terms of use, available at https://www.cambridge.org/core/terms. https://doi.org/10.1017/9781108953078

Route 3: Group-Level Effects

Group-level conflict, and the risk factors that increase its likelihood, constitute the third route in our model of how rapid global warming greatly increases aggressive and violent behavior. Many of the risk factors we have discussed previously play a significant role here as well, for example, political/economic instability, perceived inequality, and resource scarcity and their sequela. One example is the recent and currently escalating conflict over water shortages in India. In 2019, a severe drought hit one of the most populated regions in the country, causing a severe shortage in the water supply because of a delayed monsoon season. As the drought continued, protesting and rioting escalated leading to the arrests of hundreds of protestors (Neuhauser, 2019). Police were sent to guard public water supplies. Many people were injured (both police and civilians) as they fought for what little water was available (Tomlinson, 2019). It is these kinds of condition and the failure of existing institutions to adequately support people during periods like this that are ripe for an increase in conflict and violence.

Another alarming study found that a single standard deviation increase in drought intensity could increase the chances of intergroup conflict by as much as 62 percent (Maystadt & Ecker, 2014). Another study mentioned also found that a standard deviation increase in rainfall and warmer temperatures were associated with a 14 percent increase in intergroup violence (Hsiang, Burke, & Miguel, 2013). Other research suggests that these effects will be felt disproportionately by communities that are already facing poverty and other economic disadvantages (Agnew, 2011). It is clear that the communities that will be most severely affected by climate change in the short term are those that are already disadvantaged (Plante et al., 2017).

Ecomigration and Intergroup Conflict

One relatively new term illustrates the magnitude of the effects of rapid climate change on intergroup conflicts: ecomigration. *Ecomigration* refers to group migration in response to environmental, economic, or political instability brought on them by an ecological disaster (e.g., Plante & Anderson, 2017). Some ecological disasters are largely unrelated to global warming, such as volcanoes, earthquakes, and tsunamis.[3] However, many other types of severe disaster are already increasing in frequency and/or severity as a direct result of

[3] Of course, when combined with ongoing sea-level increases, tsunamis will have increasingly disastrous effects on many populations, further increasing ecomigration.

Downloaded from https://www.cambridge.org/core. Iowa State University Library, on 07 Feb 2022 at 18:20:25, subject to the Cambridge Core terms of use, available at https://www.cambridge.org/core/terms. https://doi.org/10.1017/9781108953078

human-caused global warming, such as hurricanes, tornados, coastal flooding, droughts, and wildfires.

Ecomigration does not inevitably lead to *major* intergroup conflicts. Rather, there often are additional associated risk factors that, when combined with ecomigration, almost guarantee major conflicts. For example, economic instability frequently characterizes both the region from which the ecomigrants are fleeing *and* the area to which the ecomigrants move. That means that these two groups are competing for the same scarce resources, which *usually* leads to conflict. Indeed, this competition over scarce resources is a major motivation for group conflict (regardless of whether those resources are actually scarce, or simply are *perceived* to be). Early research on the *theory of realistic group conflict* showed that previously unacquainted boys assigned to one of two groups at a summer camp would develop hostile attitudes and stereotypes towards members of the "other" group when they compete over scarce resources (Sherif et al., 1954/1961). This theory posits that much intergroup aggression can be explained by competition for scarce resources (Krahé, 2020). Even moral superiority and religious salvation can be framed as scarce resources that can belong only to one group (Avalos, 2005).

A key component that influences the outcome of intergroup contact is the "functional relationship" between two groups competing for resources (Krahé, 2020). If the relationships are positive and cooperative, then feelings about working with an outgroup to achieve a shared goal (or to acquire a shared resource) are positive. However, if the relationship is competitive, then negative stigmas, stereotypes, and prejudice about the outgroup form and the relationship can become confrontational and potentially violent (Barlow et al., 2012; Pettigrew, 2021). If the receiving governments and dominant religious, racial/ethnic groups do not want the migrants for whatever reasons (e.g., perceptions of competition over resources, loss of economic opportunity, existing hatreds, and stereotypes), then intergroup conflict becomes inevitable. In short, it is easy to see how the massive ecomigrations triggered by rapid climate change will lead to increased group conflict.

Several closely related theories (and hundreds of supporting empirical studies) further address how intergroup contact can increase hostility and violence between the groups. *Relative deprivation theory* (Smith, Pettigrew, Pippin, & Bialosiewicz, 2012) asserts that intergroup tensions arise from the *perception* that one group is given unfair resources over the other group. Group identity shapes attitudes and perceptions of ingroup and outgroup members, whether fairly or unfairly. Importantly, it is the *perceptions* of the other group that creates hostility, not necessarily the actual material conditions themselves.

Downloaded from https://www.cambridge.org/core. Iowa State University Library, on 07 Feb 2022 at 18:20:25, subject to the Cambridge Core terms of use, available at https://www.cambridge.org/core/terms. https://doi.org/10.1017/9781108953078

Intergroup threat theory (Stephan, Ybarra, & Rios Morrison, 2009) and *social identity theory* (Islam, 2014; Tajfel, 1981) note that contact with an outgroup can create a sense of threat that then leads to hostile attitudes and beliefs about the outgroup and extend the "threats" well beyond material resources. One's social identity (and sense of well-being) is closely linked to one's ingroups, including religious, political, and racial/ethnic identities. For example, if ecomigrants have a different religion than that of the receiving country, many citizens (and politicians) of that country will perceive that difference as a threat to the basic fabric of the country, regardless of whether the ecomigrants are a burden or bonus to the material well-being of the country. Hostility and prejudice towards an outgroup, such as ecomigrants, can arise merely because of preferential treatment of (and positive feelings towards) members of one's ingroups. People who strongly identify with one or more of their ingroups (that is, people whose self-worth is tied to the success of their ingroups), are especially threatened by migrants who differ on key self-identities such as nationality, race, gender, politics, and religion. Such self-identities are constantly in operation, with some more salient at some times than others.

It is easy to see how these group dynamics come into play because of rapid climate change and ecomigration. Several examples illustrate the interplay of these normal intergroup processes.

One concerns the ongoing civil war in Syria. This conflict began with an (at the time) unprecedented drought in the region (Gleick, 2014). Essentially (and this is a slight oversimplification of the mechanisms at play), this drought caused a large portion of the rural population to relocate into more urban cities in search of jobs, water, and food. Attempts at government intervention were few, ineffective, and poorly received, as the already unstable government failed to provide adequate housing, resources, and jobs for this large group of migrants. This led to increased political and civil unrest, which combined with other pre-existing conditions to help spark the eventual outbreak of civil war.

Interestingly, this conflict then led to a mass movement of Syrian groups to other countries, especially to Europe, which helped trigger tensions and anxieties about immigration.[4] This, in turn, fueled politically conservative anti-immigrant movements in Europe, such as Brexit (Einbinder, 2018; Garret, 2019; Krahé, 2020). Although there has been debate on whether the violence in Syria is directly related to climate change, this remains a perfect case study of how environmental disasters can spark eventual mass migration

[4] Simultaneous increases in immigrants from African also contributed (Krahé, 2020).

Downloaded from https://www.cambridge.org/core. Iowa State University Library, on 07 Feb 2022 at 18:20:25, subject to the Cambridge Core terms of use, available at https://www.cambridge.org/core/terms. https://doi.org/10.1017/9781108953078

that has global geopolitical consequences. For example, a similar “immigrant crisis” in the United States has been exploited by alt-right political groups, helping contribute to President Donald Trump’s election in 2016 and subsequent anti-immigrant attitudes, stereotypes, and harsh anti-immigrant laws and policies (Donovan & Redlawsk, 2018; Hesson & Kahn, 2020). In general, anti-immigrant rhetoric and policy stem mostly from an increase in support for far-right movements (Dennison & Geddes, 2018; Einbinder, 2018). This increased anxiety about immigration, the dialogue surrounding it, and policies that harm immigrants, yield a very dangerous picture for our future.

Terrorism, Terrorists, and Terrorist Groups

These same Route 3 factors play a substantial role in the development and expansion of terrorism, often interacting with Route 2 (creating violence-prone adults). The creation and expansion of terrorist groups and terrorist activities are based on rather complex relationships between humans and their environmental, economic, and social conditions (Kruglanski et al., 2009). The rapid and dramatic loss of one’s family, culture, or livelihood combined with perceptions of inequality that disproportionally harms a distinct ethnic, religious, or social group provide motivation for and success at recruitment and enlistment in terrorist groups (Goodhand, 2003; Ohlsson, 2000. This is especially true when the people that are affected believe that there is no chance at significant or substantial material improvement. Research into people joining militia groups across the globe found these factors to be the major motivations for potential recruits (Archibald et al., 2002; Hage, 2003; Maclure & Sotelo, 2004). Additionally, research on terrorism and the motivations for joining violent groups has found that not only are there material, environmental, and motivating political motives, but that individual psychological motivations – such as regaining a sense of belonging, status, power, control, and sense of meaningfulness – are major contributors as well (Goodhand, 2003; Hage, 2003; Maclure & Sotelo, 2004).

Committing acts of terror is a result of numerous societal, ideological, and personal causes (Kruglanski & Orehek, 2011). Rapid climate change has immediate and devastating effects on the most basic resources needed for global stability, and thus, increasingly large parts of the world will experience these risk factors, and, subsequently, the formation of and support for violent extremist groups will increase, especially in the most disadvantaged, unstable, and disproportionally affected countries and cultures.

Downloaded from https://www.cambridge.org/core. Iowa State University Library, on 07 Feb 2022 at 18:20:25, subject to the Cambridge Core terms of use, available at https://www.cambridge.org/core/terms. https://doi.org/10.1017/9781108953078

Additional Psychological Processes at Work

Basic Psychological Processes at Work

As mentioned earlier, one way to make a target audience feel threatened by and fearful of some outgroup is to blame "them" for the ingroup's failures, difficulties, and hardships. This is particularly effective if a solution is offered, one that targets one or more outgroups as the cause of the ingroup's difficulties. Of course, the threat does not have to be factually true. But oftentimes, highly salient events do trigger major changes in large swaths of a country's population. For example, the Islamic terrorist attack in Madrid in 2004 led to a sharp increase anti-Arab attitudes (Echebarria-Echabe & Fernández-Guede, 2006). It also led to increases in authoritarianism and anti-Semitism. Similarly, the terrorist attack of September 11, 2001, on several US targets led to more positive attitudes towards war among US college students, an increase that lasted at least a full year (Carnagey & Anderson, 2007).

The increasing hostility towards outgroups in response to the threat of climate change was being demonstrated as long as a decade ago (Fritsche, Cohrs, Kessler, & Bauer, 2012). These researchers found that merely reminding people of the adverse consequences that climate change may have for their country increased general authoritarian attitudes (which are hostile to outsiders) and derogation of outgroups.

Several common but seemingly different social-psychological effects – such as the foot-in-the-door phenomenon, conformity, compliance, and attitude change effects – help explain how rapid climate change can create and intensify outgroup hostility and aggression through a combination of Routes 2 and 3 processes. Techniques to induce specific attitudes, beliefs, and behavioral tendencies and to change these have been studied by both basic science and applied science researchers. These include hundreds of studies on stereotypes, prejudice, and racism, some dating back to World War II. Many studies have specifically focused on how humans are led to believe extreme ideologies (such as terrorist ideologies).

Early studies of mass violence focused on understanding the "authoritarian personality," hypothesized to underlie the success of Nazi ideology and of similar right-wing movements. But how do such extreme constellations of hostile attitudes, stereotypes, and behavioral inclinations develop and persist? It cannot be the case that all or even most citizens of 1930s' and early 1940s' Germany had this personality type. This belief system had to develop over time.

The *compliance* literature discovered that normal people could be induced to do violent behaviors (such as delivering potentially fatal electric shocks) against another person by first pressuring them to perform very mildly aggressive

Downloaded from https://www.cambridge.org/core. Iowa State University Library, on 07 Feb 2022 at 18:20:25, subject to the Cambridge Core terms of use, available at https://www.cambridge.org/core/terms. https://doi.org/10.1017/9781108953078

actions (e.g., a barely noticeable shock) for a "good" cause, and then gradually increasing the severity of the demanded action (e.g., Milgram, 1963). Similarly, the foot-in-the-door behavior change technique involves using social norms to induce a person to first agree to a small request, followed by subsequently larger and more intense ones (Cialdini, 2021).

The *conformity* literature has shown (among other things) that people will conform to even incorrect judgments and opinions of others, especially when those judgments are public and are somewhat ambiguous. Over time, repeated conformity to others' judgments can become internalized.

In general, the attitude change literature shows that one effective way to change attitudes is to first identify the target's "latitude of acceptance" and "latitude of rejection" on the specific attitude topic. Then, one directs persuasive communications to the target person (or group) that are in the direction of intended change but that still fall within their latitude of acceptance. Over time and repeated persuasive communications of this type, the overall attitude and the latitude of acceptance move in the targeted direction (Petty & Cacioppo, 2012).

In all cases, these techniques are commonly used to manipulate people into eventually engaging in behaviors they normally would not have engaged in, including engagement in terrorism, war, and genocide. In Nazi Germany (and other countries complicit in the Holocaust), Hitler's Reich Minister for Propaganda and Public Enlightenment, Josef Goebbels, was reasonably successful in using mass media to increase existing suspicion and hatred of Jews.

Over time, repeated use of these techniques, each of which induces small changes to existing attitudes, stereotypes, and behavioral inclinations, can eventually yield extreme beliefs, attitudes, and behaviors in the targeted population. That is, "normal" people can be changed into people (and groups) willing to join extremist groups and commit atrocities. In other words, the extremist view becomes normalized.

The process starts most often as a small concession. For instance, let us say you are frustrated with your government's perceived poor ability to distribute resources. You see that some resources are being distributed to asylum seekers, climate refugees, and immigrants, but, for some reason, not to you or your favored ingroups. The extreme ideology would start by telling you that since there is a finite number of resources, that outsiders are taking the resources *you* should have. That creates frustration at the immigration process and with the system that distributes those resources. Over time, you may slowly start to be convinced that rather than a lack of equitable distribution being a fault of the systems at play, the issue stems from *the group of people you see as a threat* to your well-being and identity. This can happen both on an individual level as

Downloaded from https://www.cambridge.org/core. Iowa State University Library, on 07 Feb 2022 at 18:20:25, subject to the Cambridge Core terms of use, available at https://www.cambridge.org/core/terms. https://doi.org/10.1017/9781108953078

well as a group level, much like we are seeing with the rise of far-right ideologies containing anti-immigration rhetoric.

Collective animosity toward outgroups manifests in other ways as well, such as in harmful representations of outgroups in news and entertainment media. This, in turn, can create feedback loops for these hostile attitudes and increase them. Frequently, hostile beliefs about and attitudes towards outgroups are not based on real-life experiences with outgroup members but rather are formed from media presentations of different groups (Nisbet, Ostman, & Shanahan, 2009; Saleem et al., 2017).

Political and Social Unrest

The crisis in Syria illustrates how rapid climate change can cause political and social unrest, but it is not the only country that has faced severe climate-related disasters that resulted in civil unrest and instability. A similar drought in Uganda created a situation in which food prices rose drastically, forcing mass migration within the country. Similar events have taken place in Kenya, Sudan, and Ethiopia, all with a clear pattern of eventually turning into a severe conflict (Plante & Anderson, 2017).

Ecological disasters directly related to climate change are not the only type that has a major impact on ecomigration and intergroup violence. In fact, there are political and social actions that can lead to these outcomes as well. One example is the recent increasing tension between Egypt, the Sudan, and Ethiopia over the potential damming of the Nile River. Ethiopia is currently constructing a dam known as the "Great Ethiopian Renaissance Dam," an attempt to provide a substantial amount of Ethiopia's growing population with more electricity (Asala, 2021). However, both Sudan and Egypt have major concerns over the dam, with Egypt's main concern being the irrigation and agricultural impacts of slowing the Nile's water flow will have on their country, and Sudan's concern about what effect the Ethiopian dam could have on its own damming infrastructure. This conflict has seen numerous attempts at peaceful resolution and could yet see a successful negotiated outcome. However, as of the time of writing, Egypt's president has mentioned that "all options are open" if Ethiopia continues to pursue the project without resolution (Reuters, 2021). Rapid climate change will require other similar major infrastructure projects in the future; this recent tension provides a good case study for how such projects can inadvertently become sources of new intergroup conflicts.

Another example has been the recent migration of 10 million people from Bangladesh into India. While this was occurring, public sentiment in India was

Downloaded from https://www.cambridge.org/core. Iowa State University Library, on 07 Feb 2022 at 18:20:25, subject to the Cambridge Core terms of use, available at https://www.cambridge.org/core/terms. https://doi.org/10.1017/9781108953078

that the migrants were responsible for stealing farmland, which led to conflict in which nearly 2,000 migrants were killed (Plante & Anderson, 2017). In the United States, Hurricane Katrina led directly to the relocation of thousands of Americans, most moving to neighboring states seeking refuge. In cities that accepted refugees (technically, *internally displaced persons*), homicide rates started to rise, creating tension between long-time residents and the refugees, in spite of the fact that there was no evidence that the increase in homicides was being committed by the new arrivals.

As noted, countries that already suffer from instability and conflict are the most vulnerable to these kinds of events. For example, it has been found that densely populated countries that are also struggling with land, crop, water, or livestock loss have been found to be even more susceptible and at risk to increased aggression and violence resulting from rapid climate change (Hallegatte et al., 2016; Mares et al., 2015; Van de Vliert, 2013).

However, this is not only true at the country level, it can also be seen within local neighborhoods. One study examined crime data in the United States city of St. Louis and found that within disadvantaged neighborhoods in the city, hot temperatures yielded a disproportional rise in crime (Mares, 2013).

The data from St. Louis shows us that even in parts of the world that are seen as more economically stable, it is those in disadvantaged communities that will experience disproportionate amounts of harm from rapid climate change (Plante et al., 2017). Furthermore, even though wealthy/stable countries have many significant protective factors, as climate change progresses, children in these countries will be exposed to more risk factors for becoming violence-prone adults (Van de Vliert, 2013). In short, *all* countries will face the consequences of rapid climate change on their own citizens. Our global infrastructure must begin to implement climate change mitigation policies, and must do so in an equitable way so as to not to continue to disproportionally harm disadvantaged communities and countries.

Housing, Climate "Gentrification," and Environmental Racism

Worldwide the most economically disadvantaged groups tend to be concentrated in the most dangerous situations (e.g., jobs, neighborhoods), where they are most likely to be immediately harmed by the effects of rapid climate change. Examining how natural disasters most often play out, it becomes clear that it is disadvantaged populations (usually racial/ethnic minorities) that suffer the most material damage, their neighborhoods suffer the most infrastructure loss, and their families are most likely to lose homes and belongings. This has been

Downloaded from https://www.cambridge.org/core. Iowa State University Library, on 07 Feb 2022 at 18:20:25, subject to the Cambridge Core terms of use, available at https://www.cambridge.org/core/terms. https://doi.org/10.1017/9781108953078

described as "environmental racism," as it looks and feels like other processes that are based on racism, such as redlining.

A strict definition of environmental racism is hard to come by, but an article by geographer Ryan Holifield (2001) used a definition we find uniquely apt. It comes from a 1982 speech by activist Benjamin Chavis:

Environmental racism is racial discrimination in environmental policy-making and enforcement of regulations and laws, the deliberate targeting of communities of color for toxic waste facilities, the official sanctioning of the presence of life-threatening [sic] poisons and pollutants for communities of color, and the history of excluding people of color from leadership of the environmental movement (Chavis, 1994, p. xii)

This definition doesn't quite capture the full extent of the discrimination one can see in environmental policies, as well as how these policies and societal structures materially impact and harm people. One exemple that highlights how some of this works is the destruction that occurred in New Orleans with Hurricane Katrina. Estimates suggest that roughly 150,000–200,000 occupied homes were destroyed or damaged when the hurricane struck (Gibb, 2016). This affected approximately 500,000–600,000 residents, displacing them from their homes anywhere from a short period of time to permanently. The adverse outcomes of this devastation have been found to have disproportionally affected communities of color and low-income homes. Survey sampling from Gallup, CNN, and others in 2005 found, that among respondents, those who identified as black were much more likely to have gone without food for at least a day (53 percent of black respondents as opposed to 24 percent for white respondents), to have been separated from their family for at least a day or more (55 percent against 45 percent), had their vehicle damaged (47 percent/ 31 percent), went without water for a day (45 percent/21 percent), and were more likely to have to spend at least one night in an emergency shelter (34 percent/13 percent). Similar discrepancies were found amongst respondents on the lines of income, with those making less than $20,000 a year also much more likely to have faced these same adversities.

In the aftermath of the hurricane, it became clear that those who were of diverse backgrounds or were already struggling economically were those most in need of assistance and were the most harmed by the hurricane. However, in the months and years following Katrina's landfall, it became apparent that those harmed communities and individuals did not receive the assistance they needed (which led to the infamous quote on live TV by rapper Kanye West, who stated that "George Bush doesn't care about black people," when participating in a fundraiser for relief funds). Even more alarming is that data from 2016

Downloaded from https://www.cambridge.org/core. Iowa State University Library, on 07 Feb 2022 at 18:20:25, subject to the Cambridge Core terms of use, available at https://www.cambridge.org/core/terms. https://doi.org/10.1017/9781108953078

show there were roughly 96,000 fewer black individuals living in New Orleans than there were before the hurricane (Rivlin, 2016). Additionally, the economic assistance that was offered and delivered, and the areas of the city that were subsequently rebuilt, were not those that saw the greatest levels of damage and relocation; rather, it was the wealthier areas who received these resources.

Low-income minority communities, such as Seventh Ward, saw minimal reconstruction and development in the aftermath of Katrina (Rivlin, 2016). A full decade after the hurricane, only 60 percent of the neighborhood was rebuilt. The Lower Ninth Ward (another low-income community) was about half of its previous population size in 2016. Discriminatory housing practices can be directly linked to findings such as black homeowners being three times more likely to experience flooding (Rivlin, 2015). In 2015 and 2016 articles, Rivlin delved into how some of these practices materialized and were implemented, explaining that because of these policies, "the high-ground was taken by the time banks (who [sic]) started loaning money to African Americans who wanted to buy a home" (Rivlin, 2016).

Other discriminatory actions were openly brazen. Rivlin noted in a *Wall Street Journal* interview that a prominent New Orleans businessman stated that the city should focus on recovery and growth in a way that would change the demographics and geopolitics of the city, or else wealthy (and mostly white) businessmen would likely abandon it. This was on top of policies implemented after the hurricane that some politicians described as "ethnic cleansing by inaction" (Mosendz, 2015; Rivlin, 2016). Rivlin's work tells more of what took place in the aftermath of the hurricane:

> Now, New Orleans no longer has a public hospital, though prior to Katrina, it was home to the nation's oldest one. Before the storm, the city was home to thousands of units of affordable housing in a quartet of housing projects locals now call the "Big Four." Large portions of the Big Four had escaped with little or no water damage. Yet elected officials chose to bulldoze all four anyway. The largest housing recovery program in U.S. history, "Road Home," was created in the months after Katrina. But money was disbursed based on the appraised value of a home rather than the cost of rebuilding, even though a home in a white community was typically appraised at a far higher price than the same house in a black community. Five years after the storm, a federal judge sided with black homeowners in a racial discrimination suit against the program. But by then, officials had already spent more than 98 percent of the $13 billion that the federal government had committed to Road Home. (Rivlin, 2016)

These actions and inactions following the hurricane greatly harmed already disadvantaged groups, making the city more difficult for them to live in. Although the extent to which the severity of Katrina can be attributed to climate

Downloaded from https://www.cambridge.org/core. Iowa State University Library, on 07 Feb 2022 at 18:20:25, subject to the Cambridge Core terms of use, available at https://www.cambridge.org/core/terms. https://doi.org/10.1017/9781108953078

change is hard to estimate, it provides a useful example that increases understanding of how many communities around the world around have been constructed and who is most immediately in harm's way from the rapid global warming. Coastal cities tend to be more urban, host more ethnic and racial diversity, and are most vulnerable to the joint effects of sea-level rise, increased storm frequency, and increased storm intensity. Previous political practices, such as those in New Orleans, set a dangerous precedent for how cities and countries respond to natural disasters, who gets the most harmed by them, and then who receives assistance and support following them. We see this manifest today in areas that have been ravaged by more recent natural disasters, such as in Puerto Rico, where many civilians still lack power years after Hurricane Maria destroyed large chunks of the island.

The recent California wildfires also provide similar insights when we see that communities of color are much more susceptible to damage from the fires and are more severely affected by the pollution that the fires cause (Davies, Haugo, Robertson, & Levin, 2018). Pair these findings with what we have discussed already about the increases in negative rhetoric towards outgroups, and we begin to see a rather bleak picture of how "climate gentrification" operates and who will suffer the most in the wake of climate change. Failing to take care of the most vulnerable communities is itself a form of violence and forcing large populations to move increases the likelihood of additional intergroup conflict, as described in earlier sections.

5 What Solutions Are There?

Four Big Steps

Considering all of this, it is easy for one to feel as if there is nothing that can be done. However, as noted in the opening section, panic is not the optimal action. Rather, all people in all societies should take their anxious energy and channel it toward finding ways to solve the multitude of issues. There are many potential solutions to the problems, no matter how scary and bleak the future may seem. The important point to remember is that there still is time to greatly mitigate the overall harm to the planet and to human civilization. There may not be much time left, but there is still time for humans to begin to right the rapid climate change ship.

We ask our readers to conder just how these individual-level factors (Routes 1 and 2) interact with the broader group-level factors (Routes 2 and 3). Beliefs, attitudes about ingroups and outgroups, and behavioral inclinations towards them are formed based on the actual material relationships between the groups *and* on the perceived relationships among those groups. The interacting perceptions of various ingroups and outgroups, in turn, can lead to conflict.

Downloaded from https://www.cambridge.org/core. Iowa State University Library, on 07 Feb 2022 at 18:20:25, subject to the Cambridge Core terms of use, available at https://www.cambridge.org/core/terms. https://doi.org/10.1017/9781108953078

Entertainment, news, and social media are where much of the action takes place. Media portrayals of certain groups can play to one's social identity, and when material resources become scarce, one can support an unequitable distribution of resources based on the salient identity groups, with a bias in favor of their own ingroup and against perceived outgroups. The basic psychological processes that underlie negative media effects can be used to shortcircuit the harmful effects and harmful feedback loops, using well-established effective methods to reduce intergroup biases and conflict (Pettigrew, 2021). That is, these same psychological principles can be used to create positive media effects (e.g., Saleem et al., 2017). We propose that figuring out how to create *holistic* conversations around these issues is the first step to take in finding potential solutions.

Step 1

The first step to take is straightforward, albeit not simple: Climate scientists, politicians, and media industries must change the rhetoric surrounding climate change. A key action is to continue to fight the battle of whether climate change is real. Climate scientists have been hard at work at this, but the combination of politicians making false claims and media leaders failing to properly identify such lies have prevented the truth from being understood and accepted by huge proportions of citizens in many countries. We are most familiar with the American situation, so our thoughts might be too focused on our own country. Nonetheless, America has contributed more to global warming (per capita) than any other country. Therefore, it is especially worrying that American politics – especially during the Trump years – has directly attacked the truth about climate change and has directly acted to thwart attempts to reign in the production of greenhouse gases. Indeed, the US pullout from the Paris accords is only a symbol of how successful they have been in hoodwinking the public. Fortunately, at the time of writing, we have a new President (Biden) and Congress who (by the barest of majorities) are trying to redirect efforts to massively reduce America's carbon footprint. Still, the outcome of the proposed legislation is very much in doubt because of continuing resistance by a sizable conservative minority of federal legislators (especially in the US Senate).

American media leaders also have failed to weed out blatant lies about climate change, giving science denialists "equal time" in news reports and in social media (e.g., Facebook, Twitter). Changes (legal ones, of course) are needed for meaningful progress to ever be made. For example, in 2018, the British Broadcasting Corporation (BBC) released a memo stating that it would no longer give airtime to climate change deniers because giving time to a clearly scientifically false position is not giving "balanced coverage."

Downloaded from https://www.cambridge.org/core. Iowa State University Library, on 07 Feb 2022 at 18:20:25, subject to the Cambridge Core terms of use, available at https://www.cambridge.org/core/terms. https://doi.org/10.1017/9781108953078

The memo stated that: "To achieve impartiality, you do not need to include outright deniers of climate change in BBC coverage, in the same way, you would not have someone denying that Manchester United won 2–0 last Saturday. The referee has spoken" (Webster, 2018). Although we believe that responsible scientists, other citizens with relevant expertise, and politicians should push back against climate science deniers, when necessary, we also believe that another (better?) approach would be like that of the BBC. Ignore the denialists and treat climate change as the problem that it is. Focus on valid science and discuss the many ways in which climate change can be reduced. That act alone by social media leaders will help guide most of the public's opinion towards accurate beliefs on the topic.

One reason for this is the assumption that to get whole societies to take significant action, people must be convinced of the scientific truths, that rapid climate change is real, is human caused, and that dramatic action will mitigate the harmful consequences. Decades of research on attitude and belief change have revealed that it is very difficult to change entrenched beliefs and attitudes. This is especially true when disinformation – such as claims that the evidence and arguments supporting the factual science are fraudulent – is constantly presented by highly visible authority figures. Humans strongly desire to protect their worldviews because changing them is psychologically costly. When their worldview is threatened, people often ignore the disconfirming evidence and arguments, downplay them, or try to dismiss them as outright lies. The psychological pressure to preserve a current worldview is dramatically increased when the new worldview is personally more threatening than the old. Not only is it hard to accept the fact that global warming presents a clear and present danger to human civilization, it is also hard to accept the fact that this impending threat is almost wholly a result of our own action and inaction. Indeed, it also may be that people simply do not perceive climate change as a major casual instigator to the material threats that they face in their daily lives. For instance, in a study conducted in Kenya, it was found that most people did not regard climate change as an immediate threat, instead focusing on other issues such as poverty, unemployment, and crime (Shisanya & Khayesi, 2007). A major challenge is getting people to understand the causal linkage between rapid global warming and these more material issues.

Step 2

The second needed step is to reframe the climate change issue, not just away from the "debate" surrounding its validity, but toward an emphasis on what

Downloaded from https://www.cambridge.org/core. Iowa State University Library, on 07 Feb 2022 at 18:20:25, subject to the Cambridge Core terms of use, available at https://www.cambridge.org/core/terms. https://doi.org/10.1017/9781108953078

both individual and societal actions are needed to mitigate harmful consequences to the planet and to human populations. Part of the hesitancy of accepting the full scope of dangers that climate change represents is that so far, much rhetoric around solutions to climate change is framed in terms of individual action. Common suggestions usually focus on using more public transit, eating less meat, recycling more, using less plastic, buying more efficient vehicles, and so on. Such appeals are useful, but when this individual framework is the primary one, other well-known psychological phenomena such as *diffusion of responsibility* and *social loafing* effects reduce the likelihood of major behavioral action. Indeed, such an exclusive focus on individual action is also personally threatening and somewhat overwhelming. The daily lives of most humans across the globe are filled with personal stressors and complex situations that demand their immediate attention, leaving many people unwilling or unable to take on the responsibility of saving the planet. The natural inclination is to assume that climate change cannot be *that* bad. Otherwise, we are doomed. Increasing public discourse on ways that governments can and should address rapid climate change will have two important consequences: (1) it relieves some of the pressure to resist changing one's old worldview to a new one that accepts the scientific truths about climate change, thereby increasing public support for taking decisive action; (2) it provides a simple action that individuals can do to address this existential threat of climate change, namely, voting for politicians who strongly advocate government actions to combat the problem, and voting against politicians who deny the need for change and/or who oppose government action.

Step 3

Successfully changing people's worldview to a more accurate one that includes the need for individual action is a major step in the right direction. But an additional step is needed. The need arises from the perception that either the crisis is not as severe as it truly is or that the actions being proposed cannot possibly be sufficient enough to deal with it. The third needed step begins with the observations that most of the harmful greenhouse gases put into the atmosphere by humans come from global corporations and a super minority of individuals. In 2017, the Carbon Majors Database published a report that detailed how just 100 corporations contribute 71 percent of the global emissions. Therefore, it seems that a much more efficient and effective way to start the global reform to reach net-zero emissions needs to be set at this higher level, not exclusively at an individual level. If legislators, journalists, and the public

Downloaded from https://www.cambridge.org/core. Iowa State University Library, on 07 Feb 2022 at 18:20:25, subject to the Cambridge Core terms of use, available at https://www.cambridge.org/core/terms. https://doi.org/10.1017/9781108953078

discourse turned its attention on policies addressed to combat the emissions caused by industry, then the crisis suddenly feels (and is) manageable.

The third step, then, is to focus public discourse more on major and tangible sources of greenhouse gas emissions *and* on tangible governmental/societal changes that drastically reduce the emissions from these sources. Rather than focusing the blame and responsibility for change on the shoulders of individuals, this approach identifies a tangible entity with material ways to adopt sweeping change.

Note that we do not advocate "blaming" the corporate sources of greenhouse gases. Doing so would trigger defensive actions (psychological, political, and legal) by people who run these corporations and those whose livelihood depends on them. Instead, public discourse should focus on encouraging businesses (especially, but not exclusively, the top 100 emitters) and governments to work cooperatively in reducing the net emissions of greenhouse gases. In short, the goal is to create a new ingroup that includes businesses, government, and individuals to create effective climate change reforms. In short, taking effective action must become more *holistic*.

Step 4

Steps 1–3 focused on addressing rapid climate change itself. That is certainly a top priority. However, we cannot help but be alarmed at the lack of public discourse addressing the secondary effects of climate change, namely, the harmful effects it will have on humans, as exemplified by the direct and indirect effects displayed in Figure 1. The main points of Steps 1–3 apply equally to these *human* issues.

Our research team first attempted to draw attention to these issues in 1998 (Anderson & Bushman) and has continued to do so in periodic articles, chapters, and encyclopedia entries (Anderson, 2001, 2012; Anderson & DeLisi, 2011; Miles-Novelo & Anderson, 2019; Plante, Allen, & Anderson, 2017; Plante & Anderson, 2017). These efforts may have influenced the thinking of some academics, but they have had little (or no) impact on public discourse. A primary goal in writing this Element is to educate a much broader audience on the dire need to address both the climate change problem and the associated human issues, using advances in the behavioral sciences.

Further Suggestions

We have outlined in this Element some of the ways that rapid climate change will fundamentally alter human behavior in harmful ways. We have also

Downloaded from https://www.cambridge.org/core. Iowa State University Library, on 07 Feb 2022 at 18:20:25, subject to the Cambridge Core terms of use, available at https://www.cambridge.org/core/terms. https://doi.org/10.1017/9781108953078

described how fundamental behavioral science principles can be used to promote successful actions designed to mitigate climate change itself and to ameliorate human problems. We have given global suggestions on how both types of issue can be addressed by effective, proactive, and holistic policy. This section outlines more specific actions.

Consider the impacts of mass ecomigration. Scientists know that hundreds of millions, if not billions, of people will be displaced from their homes because of rapid climate change. Nations of the world, especially those with substantial resources, must immediately begin drafting global policies about how to humanely, and safely, handle those who will be seeking refuge (i.e., refugees and internally displaced persons). The United Nations, the World Health Organization, and other international organizations must play major roles as well. All such organizations need to begin setting aside resources, building contingency plans, and constructing the infrastructure needed to help make sure that those fleeing newly uninhabitable regions have safe and secure places to go to. If there continues to be a lack of clear, humane, and thorough policy, then the ecomigration situation will create new breeding grounds for frustration, conflict, terrorism, and war (and expand those that already exist). The consequences of large anti-immigrant policies and hostile beliefs about ecomigrant groups will have devastating repercussions, both for those who seek refuge and for the countries that reject them. As resources become scarcer and the situation becomes more dire, a lack of effective, humane policies on handling ecomigrants invites further development of dangerous stereotypes, ideologies, and beliefs designed by the human mind to justify the inhumane treatment and neglect for those who need help.

One promising example of holistic policy surrounding climate change was the recent Green New Deal Resolution written by the United States Congresswoman Alexandria-Ocasio Cortez and Senator Ed Markey. Although the specifics are clearly US-centric, the basic ideas can be applied to other nations and international organizations. The main goal of this resolution was to suggest a rough outline of a series of policy goals that could serve as the basis for a "10-year mobilization" (Rizzo, 2019). The idea was that by creating a list of specific goals for a specified time period, US politicians could then not only focus on starting to create more substantive policies, but also find meaningful ways to accomplish them. Although the resolution calls for many of the goals we have come to associate with most climate-change proposals (such as ending carbon emissions from transportation), it also calls for the passing of legislation one may not associate with climate change, such as:

Downloaded from https://www.cambridge.org/core. Iowa State University Library, on 07 Feb 2022 at 18:20:25, subject to the Cambridge Core terms of use, available at https://www.cambridge.org/core/terms. https://doi.org/10.1017/9781108953078

passing universal healthcare making public universities tuition free providing a "federal jobs guarantee," as well as raising the federal minimum wage making housing, clean water, and access to affordable food, human rights,

Policies like this are critically important for several reasons, mainly because they address climate change itself and because they help combat the negative impacts on human societies. For example, climate change is likely to make severe outbreaks of diseases more common (again, as illustrated by the COVID-19 pandemic); one way to help combat this issue is to guarantee accessible and affordable healthcare to everyone, as well as increasing funding to public hospitals and research centers, to help combat, contain, and treat these diseases as they arise.

Many people will lose their neighborhoods to natural disasters, so having governmental housing programs to shelter those who have been displaced, as well as having a governmental job guarantee to employ those who need work, will greatly mitigate all sorts of human problems. If you recall our discussion on risk factors for aggression and violence, the stress, anxiety, and threat of losing your home and job are some of the risk factors in potential future violence. By reducing the harmful impacts that people face in the wake of climate change, we also reduce the prevalence and intensity of these risk factors, thereby reducing many of the eventual negative outcomes (e.g., creating violence-prone adults). Additionally, such actions reduce the chances of major political and social instability.

Mass media – including news, entertainment, and social media – have a major role to play in saving the planet and its people. From our US-centric perspective, change to media policies and practices is an especially important and difficult problem. In our view, all mass media have failed to effectively deal with science denialists, especially with lies promoted by major political leaders and their followers. Some of these potential actions will be difficult for political, economic, and/or legal reasons, especially in the US:

Correct the blind policy of giving equal weight to vastly unequal "sides" on every issue. As the BBC has done, take into account that demonstrable falsehoods (scientific or other) do not warrant the same free speech protections as demonstrable truths or reasonably debatable issues. Keep in mind that the US Constitution implies that everyone is entitled to their own opinion. People are not entitled to their own "facts." Other countries may have similar political/legal issues to handle.

Invest in creating and distributing entertaining (and thereby profitable) media that accurately portray the climate change issues and that do so in ways designed to promote effective action at both the individual level and

Downloaded from https://www.cambridge.org/core. Iowa State University Library, on 07 Feb 2022 at 18:20:25, subject to the Cambridge Core terms of use, available at https://www.cambridge.org/core/terms. https://doi.org/10.1017/9781108953078

the societal level. This could be done in films (e.g., *The Day After Tomorrow*, 2004), documentaries, television, internet series, and video games.

Create and frequently air persuasive (and nonthreatening) public service announcements. US television already is required to do PSAs as part of their licensing requirements. These brief announcements should be used to educate the public about this most important human crisis. Doing so will also help counter the disinformation.

Clarify and relabel what constites “news” shows. For instance, do not allow shows that consistently fail to meet journalistic standards for accuracy to be labeled or marketed as news (e.g., Fox News); they might better be labeled as entertainment and be required to state that they often contain false information, like the required warning labels on tobacco products. We suspect that if the mass media in the US does not do so, then government intervention will become more likely.

6 Conclusions

We believe that progressive, proactive, and holistic policies offer something that is crucial to handling the climate change crisis: hope. A common therapeutic technique used by psychologists is to have clients reframe the issues they are having. They suggest looking for ways to put positive and constructive energy towards creating solutions, rather than focusing primarily on potentially negative outcomes or on blaming themselves for past failures (real, exaggerated, or wholly imagined). By offering policies that not only address human-caused climate change, but that also address human problems arising from climate change, the world can start to piece together effective problem-solving systems.

The specific approaches suggested throughout this Element additionally benefit from several basic psychological phenomena. For example, the *repetition effect* demonstrates that the more often people hear/see a “fact,” the more accepting they become of that fact. This is one reason why we so strongly advocate for policies that reduce people’s exposure to claims that are demonstrably false.

Research on *behavioral script development and change* demonstrates that the more frequently that people think about or imagine a sequence of events that lead to a specific outcome, the more they believe the sequence to be attainable and the more they expect the script’s outcome to occur. Such scripts are highly integral to human beliefs, motivations, and behaviors.

The advocated approaches will also lead to increased engagement in *social comparison* on climate change beliefs and attitudes. Social comparison is the pervasive process through which people observe their peers to assess the

Downloaded from https://www.cambridge.org/core. Iowa State University Library, on 07 Feb 2022 at 18:20:25, subject to the Cambridge Core terms of use, available at https://www.cambridge.org/core/terms. https://doi.org/10.1017/9781108953078

validity of their own attitudes, beliefs, and actions. Such comparisons can lead to change, especially when the comparison targets are admired and seen as experts.

Mechanisms that underlie *social facilitation* and *conformity* also can be used to promote belief and behavioral change in the desired direction of accuracy. Social facilitation occurs when people increase their efforts on a task and is most likely to occur when they see others performing the desired action and feel that they are accountable to behave in a similar way. Conformity to the attitudes and beliefs of others in their social environment is a routine human phenomenon that occurs through several cognitive and dynamic group processes beyond the scope of this monograph. Such processes help explain why many can still resist the scientific facts about climate change and about COVID-19. Following our suggestions about reframing the issues, decreasing exposure to falsehoods, and increasing exposure to the facts in nonthreatening ways will increase the general public's understanding of, belief in, and attitudes towards the positive actions needed to handle this crisis.

There is no one solution to the climate change problems. Nonetheless, we believe (as do most behavioral scientists with whom we have communicated) that reframing the problems using the approaches that we derived from the behavioral sciences will have a tremendous positive impact on our planet and the people on it. We hasten to add that the ideas we have presented in this Element are not unique to us but rather have come from many scholars, past and present, whom we cannot credit without severely overstepping the very reasonable page and reference limitations of this Element.

Downloaded from https://www.cambridge.org/core. Iowa State University Library, on 07 Feb 2022 at 18:20:25, subject to the Cambridge Core terms of use, available at https://www.cambridge.org/core/terms. https://doi.org/10.1017/9781108953078

References

Adams, D. (1980). *The Hitchhiker's Guide to the Galaxy* (BCA Edition). London: Book Club Associates.

Agnew, J. (2011). Waterpower: Politics and the geography of water provision. *Annals of the Association of American Geographers*, *101*, 463–76.

Akbarzadeh, S. & Smith, B. (2005). *The Representation of Islam and Muslims in the Media:* The Age *and* Herald Sun *Newspapers*. Clayton, VIC: School of Political and Social Inquiry, Monash University.

Alsultany, E. (2012). *Arabs and Muslims in the Media Race and Representation after 9/11*. New York: New York University Press.

Anderson, C. A. (1989). Temperature and aggression: Ubiquitous effects of heat on the occurrence of human violence. *Psychological Bulletin*, *106*, 74–96.

Anderson, C. A. (2001). Heat and violence. *Current Directions in Psychological Science*, *10*, 33–8.

Anderson, C. A. (2012). Climate change and violence. In D. J. Christie (ed.), *The Encyclopedia of Peace Psychology* (pp. 128–32). Hoboken, NJ: Wiley-Blackwell.

Anderson, C. A. & Anderson, D. C. (1984). Ambient temperature and violent crime: Tests of the linear and curvilinear hypotheses. *Journal of Personality and Social Psychology*, *46*, 91–7.

Anderson, C. A. & Anderson, K. B. (1996). Violent crime rate studies in philosophical context: A destructive testing approach to heat and southern culture of violence effects. *Journal of Personality and Social Psychology*, *70*, 740–56.

Anderson, C. A. & Anderson, K. B. (1998). Temperature and aggression: Paradox, controversy, and a (fairly) clear picture. In R. Geen & E. Donnerstein (eds.), *Human Aggression: Theories, Research, and Implications for Social Policy* (pp. 247–98). San Diego, CA: Academic Press.

Anderson, C. A., Anderson, K. B., & Deuser, W. E. (1996). Examining an affective aggression framework: Weapon and temperature effects on aggressive thoughts, affect, and attitudes. *Personality and Social Psychology Bulletin*, *22*, 366–76.

Anderson, C. A., Anderson, K. B., Dorr, N., DeNeve, K. M., & Flanagan, M. (2000). Temperature and aggression. *Advances in Experimental Social Psychology*, *32*, 63–133.

Anderson, C. A., Buckley, K. E., & Carnagey, N. L. (2008). Creating your own hostile environment: A laboratory examination of trait aggression and the

Downloaded from https://www.cambridge.org/core. Iowa State University Library, on 07 Feb 2022 at 18:20:25, subject to the Cambridge Core terms of use, available at https://www.cambridge.org/core/terms. https://doi.org/10.1017/9781108953078

violence escalation cycle. *Personality and Social Psychology Bulletin*, *34*, 462–73.

Anderson, C. A. & Bushman, B. J. (1998). Will global warming inflame our tempers? *APA Monitor, 49 #2*, February issue.

Anderson, C. A. & Bushman, B. J. (2002). Human aggression. *Annual Review of Psychology*, *53*, 27–51.

Anderson, C. A., Bushman, B. J., & Groom, R. W. (1997). Hot years and serious and deadly assault: Empirical tests of the heat hypothesis. *Journal of Personality and Social Psychology*, *73*, 1213–23.

Anderson, C. A. & Carnagey, N. L. (2004). Violent evil and the general aggression model. In A. Miller (ed.), *The Social Psychology of Good and Evil* (pp. 168–92). New York: Guilford Publications.

Anderson, C. A. & DeLisi, M. (2011). Implications of global climate change for violence in developed and developing countries. In J. Forgas, A. Kruglanski, & K. Williams (eds.), *The Psychology of Social Conflict and Aggression* (pp. 249–65). New York: Psychology Press.

Anderson, C. A., Deuser, W. E., & DeNeve, K. (1995). Hot temperatures, hostile affect, hostile cognition, and arousal: Tests of a general model of affective aggression. *Personality and Social Psychology Bulletin*, *21*, 434–48.

Anderson, C. A. & Kellam, K. L. (1992). Belief perseverance, biased assimilation, and covariation detection: The effects of hypothetical social theories and new data. *Personality and Social Psychology Bulletin*, *18*, 555–65.

Anderson, C. A. & Lindsay, J. J. (1998). The development, perseverance, and change of naive theories. *Social Cognition*, *16*, 8–30.

Archibald, S. & Richards, P. (2002). Converts to human rights? Popular debate about war and justice in rural Sierra Leone. *Africa*, *72*, 339–67.

Asala, K. (2021, April 8). Tension over Nile waters between Ethiopia, Sudan and Egypt explained. Africanews. www.africanews.com/2021/04/08/tension-over-nile-waters-between-ethiopia-sudan-and-egypt-explained//.

Asch, S. E. (1952/1972). Group forces in the modification and distortion of judgments. In E. P. Hollander & R. G. Hunt (eds.), *Classic Contributions to Psychology* (pp. 330–9). New York: Oxford University Press.

Auliciems, A. & DiBartolo, L. (1995). Domestic violence in a subtropical environment: Police calls and weather in Brisbane. *International Journal of Biometeorology*, *39*(1), 34–9.

Avalos, H. (2005). *Fighting Words: The Origins of Religious Violence*. Amherst, NY: Prometheus Books.

Barlow, F. K., Paolini, S., Pedersen, A. et al. (2012). The contact caveat: Negative contact predicts increased prejudice more than positive contact

Downloaded from https://www.cambridge.org/core. Iowa State University Library, on 07 Feb 2022 at 18:20:25, subject to the Cambridge Core terms of use, available at https://www.cambridge.org/core/terms. https://doi.org/10.1017/9781108953078

predicts reduced prejudice. *Personality and Social Psychology Bulletin*, *38*, 1629–43.

Barnett, J. & Adger, W. (2007). Climate change, human security and violent conflict. *Political Geography*, *26*, 639–55.

Behm-Morawitz, E. & Ortiz, M. (2013). Race, ethnicity, and the edia. In K. Dill (ed.), *The Oxford Handbook of Media Psychology* (pp. 252–64). New York: Oxford University Press.

Berkowitz, L. (1989). Frustration-aggression hypothesis: Examination and reformulation. *Psychological Bulletin*, *106*, 59–73.

Boulant, J. A. (1981). Hypothalamic mechanisms in thermoregulation. Federation Proceedings. December, *40*(14), 2843–50.

Boyanowsky, E. O. (1999). Violence and aggression in the heat of passion and in cold blood. *International Journal of Law and Psychiatry*, *22*, 257–71.

Boyanowsky, E. O. (2008). Explaining the relation among environmental temperatures, aggression and violent crime: Emotional-cognitive stress under thermoregulatory conflict (ECS-TC Syndrome). Presented at the Biannual World Meeting of the International Society for Research on Aggression, Budapest, Hungary, July 8–13.

Boyanowsky, E. O., Calvert-Boyanowsky, J., Young, J., & Brideau, L. (1981). Toward a thermoregulatory model of violence. *Journal of Environmental Systems*, *11*, 81–7.

Bushman, B. J., Coyne, S. M., Anderson, C. A. et al. (2018). Risk factors for youth violence: Youth Violence Commission, International Society for Research on Aggression. *Aggressive Behavior*, *44*, 331–6.

Bushman, B. J., Wang, M. C., & Anderson, C. A. (2005a). Is the curve relating temperature to aggression linear or curvilinear? Assaults and temperature in Minneapolis reexamined. *Journal of Personality and Social Psychology*, *89*, 62–6.

Bushman, B. J., Wang, M. C., & Anderson, C. A. (2005b). Is the curve relating temperature to aggression linear or curvilinear? A response to Bell (2005) and to Cohn and Rotton (2005). *Journal of Personality and Social Psychology*, *89*, 74–7.

Carlsmith, J. M. & Anderson, C. A. (1979). Ambient temperature and the occurrence of collective violence: A new analysis. *Journal of Personality and Social Psychology*, *37*, 337–44.

Carnagey, N. L. & Anderson, C. A. (2007). Changes in attitudes towards war and violence after September 11, 2001. *Aggressive Behavior*, *33*, 118–29.

Caspi, A., McClay, J., Moffitt, T. E. et al. (2002). Role of genotype in the cycle of violence in maltreated children. *Science*, *297*(5582), 851–4.

Downloaded from https://www.cambridge.org/core. Iowa State University Library, on 07 Feb 2022 at 18:20:25, subject to the Cambridge Core terms of use, available at https://www.cambridge.org/core/terms. https://doi.org/10.1017/9781108953078

Chang, C. H., Bernard, T. E., & Logan, J. (2017). Effects of heat stress on risk perceptions and risk taking. *Applied Ergonomics*, *62*, 150–7.

Chavis, B. F., Jr. (1994). Preface. In R. D. Bullard (ed.), *Unequal Protection: Environmental Justice and Communities of Color* (pp. xi–xii). San Francisco, CA: Sierra Club Books.

Chen, E., Cohen, S., & Miller, G. E. (2010). How low socioeconomic status affects 2-year hormonal trajectories in children. *Psychological Science*, *21*, 31–7.

Cialdini, R. B. (2021). *Influence, New and Expanded: The Psychology of Persuasion*. New York: HarperCollins.

Cohen, D. & Nisbett, R. E. (1994). Self-protection and the culture of honor: Explaining southern violence. *Personality & Social Psychology Bulletin*, *20*, 551–67.

Cohen, L. E. & Felson, M. (1979). Social change and crime rate trends: A routine activity approach. *American Sociological Review*, *44*, 588–608.

Coll, C. G., Patton, F., Marks, A. K. et al. (2012). Understanding the immigrant paradox in youth: Developmental and contextual considerations. In A. S. Masten, K. Liebkind, & Hernandez, D. J. (eds.), *Capitalizing on Migration. The Potential of Immigrant Youth*. Cambridge: Cambridge University Press.

Craigie, T. A. L., Brooks-Gunn, J., & Waldfogel, J. (2012). Family structure, family stability and outcomes of five-year-old children. *Families, Relationships and Societies*, *1*(1), 43–61.

Cramer, C. (2003). Does inequality cause conflict? *Journal of International Development*, *15*, 397–412.

Daneman, R. & Prat, A. (2015). The blood–brain barrier. *Cold Spring Harbor Perspectives in Biology*, *7*(1), a020412.

Das, E., Bushman, B. J., Bezemer, M. D., Kerkhof, P., & Vermeulen, I. E. (2009). How terrorism news reports increase prejudice against outgroups: A terror management account. *Journal of Experimental Social Psychology*, *45*, 453–9.

Data Center. (2015). Who lives in New Orleans and metro parishes now? www.datacenterresearch.org/data-resources/who-lives-in-new-orleans-now/?utm_source=newsletter&utm_medium=email&utm_content=http%3A//www.datacenterresearch.org/data-resources/who-lives-in-new-orleans-now/&utm_campaign=Who%20Lives.

Davies, I. P., Haugo, R. D., Robertson, J. C., & Levin, P. S. (2018). The unequal vulnerability of communities of color to wildfire. *PloS one*, *13*(11), e0205825.

Downloaded from https://www.cambridge.org/core. Iowa State University Library, on 07 Feb 2022 at 18:20:25, subject to the Cambridge Core terms of use, available at https://www.cambridge.org/core/terms. https://doi.org/10.1017/9781108953078

Davies, K., Tropp, L. R., Aron, A., Pettigrew, T. F., & Wright, S. C. (2011). Cross-group friendships and intergroup attitudes: A meta-analytic review. *Personality and Social Psychology Review*, *15*(4), 332–51.

De Bellis, M. D. (2005). The psychobiology of neglect. *Child Maltreatment*, *10* (2), 150–72.

Deleu, D., El Siddig, A., Kamran, S. et al. (2005). Downbeat nystagmus following classical heat stroke. *Clinical Neurology and Neurosurgery*, *108* (1), 102–4.

DeLisi, M. (2005). *Career Criminals in Society*. Thousand Oaks, CA: Sage.

Dematte, J. E., O'Mara, K., Buescher, J. et al. (1998). Near-fatal heat stroke during the 1995 heat wave in Chicago. *Annals of Internal Medicine*, *129*(3), 173–81.

Dennison, J. & Geddes, A. (2018). Brexit and the perils of "Europeanised" migration. *Journal of European Public Policy*, *25*(8), 1137–53.

Dill, K. E., Gentile, D. A., Richter, W. A., & Dill, J. C. (2005). Violence, sex, race and age in popular video games: A content analysis. In E. Cole & J. Henderson Daniel (eds.), *Featuring Females: Feminist Analyses of the Media* (pp. 115–30). Washington, DC: American Psychological Association.

Dixon, T. L. & Azocar, C. L. (2007). Priming crime and activating blackness: Understanding the psychological impact of the overrepresentation of blacks as lawbreakers on television news. *Journal of Communication*, *57*, 229–53.

Doherty, T. J. & Clayton, S. (2011). The psychological impacts of global climate change. *American Psychologist*, *66*(4), 265–76.

Donovan, T. & Redlawsk, D. (2018). Donald Trump and right-wing populists in comparative perspective. *Journal of Elections, Public Opinion and Parties*, *28*(2), 190–207.

Echebarria, Echabe, A. & Fernández, Guede, E. (2006). Effects of terrorism on attitudes and ideological orientation. *European Journal of Social Psychology*, *36*(2), 259–65.

Eigsti, I. M. & Cicchetti, D. (2004). The impact of child maltreatment on expressive syntax at 60 months. *Developmental Science*, *7*(1), 88–102.

Einbinder, N. (2018). How the far right has reshaped the refugee debate in Europe. PBS. www.pbs.org/wgbh/frontline/article/how-the-far-right-hasreshaped-the-refugee-debate-ineurope/.

Eisen, M. L., Goodman, G. S., Qin, J., Davis, S., & Crayton, J. (2007). Maltreated children's memory: Accuracy, suggestibility, and psychopathology. *Developmental Psychology*, *43*(6), 1275.

Fagan, B. (2000). *The Little Ice Age: How Climate Made History 1300–1850*. New York: Basic Books.

Downloaded from https://www.cambridge.org/core. Iowa State University Library, on 07 Feb 2022 at 18:20:25, subject to the Cambridge Core terms of use, available at https://www.cambridge.org/core/terms. https://doi.org/10.1017/9781108953078

Fritsche, I., Cohrs, J. C., Kessler, T., & Bauer, J. (2012). Global warming is breeding social conflict: The subtle impact of climate change threat on authoritarian tendencies. *Journal of Environmental Psychology*, *32*(*1*), 1–10.

Gallup, Inc. (2018, November 14). Katrina hurt blacks and poor victims most. Gallup.com. https://news.gallup.com/poll/19405/katrina-hurt-blacks-poor-victims-most.aspx.

Garret, A. (2019). The refugee crisis, Brexit, and the reframing of immigration in Britain. *Europe Now*. www.europenowjournal.org/2019/09/09/the-refugee-crisis-brexitand-the-reframing-of-immigration-in-britain/.

Gibb, S. (2016, July 23). After Hurricane Katrina: Where are they now? United States Census Bureau. www.census.gov/newsroom/blogs/random-samplings/2016/05/after-hurricane-katrina-where-are-they-now.html.

Gilens, M. (1996). Race and poverty in America: Public misperceptions and the American news media. *Public Opinion Quarterly*, *60*(4), 515–41.

Gilliam Jr, F. D. & Iyengar, S. (2000). Prime suspects: The influence of local television news on the viewing public. *American Journal of Political Science*, *44*(3), 560–73.

Gilliam Jr, F. D., Valentino, N. A., & Beckmann, M. N. (2002). Where you live and what you watch: The impact of racial proximity and local television news on attitudes about race and crime. *Political Research Quarterly*, *55*(4), 755–80.

Gleick, P. H. (2014). Water, drought, climate change, and conflict in Syria. *Weather, Climate, and Society*, *6*(3), 331–40.

Goodhand, J. (2003). Enduring disorder and persistent poverty: A review of linkages between war and chronic poverty. *World Development*, *31*, 629–46.

Gramlich, J. (2020). What the data says (and doesn't say) about crime in the United States. Pew Research Center. www.pewresearch.org/fact-tank/2020/11/20/facts-about-crime-in-the-u-s/.

Griffin, P. (2017). The Carbon Majors Database CDP: Carbon Majors report 2017. Carbon Majors Database. https://b8f65cb373b1b7b15feb-c70d8ead6ced550b4d987d7c03fcdd1d.ssl.cf3.rackcdn.com/cms/reports/documents/000/002/327/original/Carbon-Majors-Report-2017.pdf?1499691240.

Hage, G. (2003). "Comes a time we are all enthusiasm": Understanding Palestinian suicide bombers in times of exighophobia. *Public Culture*, *15*, 65–89.

Hallegatte, S., Rogelj, J., Allen, M. et al. (2016). Mapping the climate change challenge. *Nature Climate Change*, *6*(7), 663–8.

Harries, K. D. & Stadler, S. J. (1983). Determinism revisited: Assault and heat stress in Dallas, 1980. *Environment & Behavior*, *15*, 235–56.

Downloaded from https://www.cambridge.org/core. Iowa State University Library, on 07 Feb 2022 at 18:20:25, subject to the Cambridge Core terms of use, available at https://www.cambridge.org/core/terms. https://doi.org/10.1017/9781108953078

Harvard T.H. Chan School of Public Health. (2020). Coronavirus and climate change. www.hsph.harvard.edu/c-change/subtopics/coronavirus-and-climate-change/.

Herring, S. C., Hoerling, M. P., Kossin, J. P., Peterson, T. C., & Stott, P. A., (eds.) (2015). Explaining extreme events of 2014 from a climate perspective. *Bulletin of the American Meteorological Society*, *96*(12), S1–S172.

Hesson, T. & Kahn, C. (2020, August 14). Trump pushes anti-immigrant message even as coronavirus dominates campaign. Reuters. www.reuters.com/article/us-usa-election-immigration-insight/trump-pushes-anti-immigrant-message-even-as-coronavirus-dominates-campaign-idUSKCN25A18W.

Holifield, R. (2001). Defining environmental justice and environmental racism. *Urban Geography*, *22*(1), 78–90.

Hsiang, S. M., Burke, M., & Miguel, E. (2013). Quantifying the influence of climate on human conflict. *Science*, *341*(6151), 1–14.

Huston, A. C. & Bentley, A. (2009). Human development in societal context. *Annual Review of Psychiatry*, *61*, 411–37.

Intergovernmental Panel on Climate Change. (IPCC). (2007). Parry, M. L., Canziani, O. F., Paultikof, J. P., van der Linden, P. J., & Hanson, C. E. (eds.), *Intergovernmental Panel on Climate Change*. Cambridge: Cambridge University Press.

Intergovernmental Panel on Climate Change. (IPCC). (2013). Parry, M. L., Canziani, O. F., Paultikof, J. P., van der Linden, P. J., & Hanson, C. E. (eds.), *Intergovernmental Panel on Climate Change*. Cambridge: Cambridge University Press.

Islam, G. (2014). Social identity theory. *Journal of Personality and Social Psychology*, *67*, 741–63.

Kalkan, K. O., Layman, G. C., & Uslaner, E. M. (2009). “Bands of others”? Attitudes toward Muslims in contemporary American society. *Journal of Politics*, *71*(3), 847–62.

Kenrick, D. T. & MacFarlane, S. W. (1986). Ambient temperature and horn honking: A field study of the heat/aggression relation. *Environmental Behavior*, *18*, 179–91.

Kovats, R. S. & Hajat, S. (2008). Heat stress and public health: A critical review. *Annual Review of Public Health*, *29*, 41–55.

Krahé, B. (2020). *The Social Psychology of Aggression*. London: Routledge.

Kruglanski, A. W., Chen, X., Deschesne, M., Fishman, S., & Orehek, E. (2009). Fully committed: Suicide bombers’ motivation and the quest for personal significance. *Political Psychology*, *30*, 331–57.

Downloaded from https://www.cambridge.org/core. Iowa State University Library, on 07 Feb 2022 at 18:20:25, subject to the Cambridge Core terms of use, available at https://www.cambridge.org/core/terms. https://doi.org/10.1017/9781108953078

Kruglanski, A. W. & Orehek, E. (2011). The role of the quest for personal significance in motivating terrorism. In J. Forgas, A. Kruglanski, & K. Williams (eds.), *The Psychology of Social Conflict and Aggression* (pp. 153–66). New York: Psychology Press

Kunda, Z. (2001). Hot cognition: The impact of motivation and affect on judgment. In *Social Cognition: Making Sense of People*. Cambridge, MA: MIT Press.

Kuwabara, M. & Smith, L. B. (2012). Cross-cultural differences in cognitive development: Attention to relations and objects. *Journal of Experimental Child Psychology*, *113*(1), 20–35.

Liu, J., Raine, A., Venables, P. H., & Mednick, S. A. (2004). Malnutrition at age 3 years and externalizing behavior problems at ages 8, 11, and 17 years. *American Journal of Psychiatry*, *161*, 2005–13.

Maclure, R. & Sotelo, M. (2004). Youth gangs in Nicaragua: Gang membership as structured individualization. *Journal of Youth Studies*, *7*, 417–32.

Mares, D. (2013). Climate change and levels of violence in socially disadvantaged neighborhood groups. *Journal of Urban Health: Bulletin of the New York Academy of Medicine*, *90*(4), 768–83.

Mares, D. M. & Moffett, K. W. (2015). Climate change and interpersonal violence: A "global" estimate and regional inequities. *Climatic Change*, online publication.

Massen, J. J., Dusch, K., Eldakar, O. T., & Gallup, A. C. (2014). A thermal window for yawning in humans: Yawning as a brain cooling mechanism. *Physiology & Behavior*, *130*, 145–8.

Maystadt, J.-F. & Ecker, O. (2014). Extreme weather and civil war: Does drought fuel conflict in Somalia through livestock price shocks? *American Journal of Agricultural Economics*, *96*(4), 1157–82.

McClure, E. S., Vasudevan, P., Bailey, Z., Patel, S., & Robinson, W. R. (2020). Racial capitalism within public health—how occupational settings drive COVID-19 disparities. *American Journal of Epidemiology*, *189*(11), 1244–53.

McCoy, K., Tibbs, J. J., DeKraai, M., & Hansen, D. J. (2020). Household dysfunction and adolescent substance use: Moderating effects of family, community, and school support. *Journal of Child & Adolescent Substance Abuse*, *29*(1), 68–79.

Miles-Novelo, A. & Anderson, C. A. (2019). Climate change and psychology: Effects of rapid global warming on violence and aggression. *Current Climate Change Reports*, *5*(1), 36–46.

Milgram, S. (1963). Behavioral study of obedience. *Journal of Abnormal and Social Psychology*, *67*(4), 371.

Downloaded from https://www.cambridge.org/core. Iowa State University Library, on 07 Feb 2022 at 18:20:25, subject to the Cambridge Core terms of use, available at https://www.cambridge.org/core/terms. https://doi.org/10.1017/9781108953078

Miller, N., Pedersen, W. C., Earleywine, M., & Pollock, V. E. (2003). A theoretical model of triggered displaced aggression. *Personality and Social Psychology Review*, *7*, 75–97.

Mosendz, P. (2016, April 14). After Hurricane Katrina, a man-made disaster in New Orleans. Newsweek. www.newsweek.com/2015/08/28/hurricane-katrina-new-orleans-rebuilding-364051.html.

Nafziger, E. & Auvinen, J. (2002). Economic development, inequality, war, and state violence. *World Development*, *30*, 153–63.

Neugebauer, R., Hoek, H. W., & Susser, E. (1999). Prenatal exposure to wartime famine and development of antisocial personality disorder in early adulthood. *Journal of the American Medical Association*, *282*, 455–62.

Neuhauser, A. (2019). Hundreds of protesters arrested in India after water runs dry. U.S. News & World Report. www.usnews.com/news/world-report/articles/2019–06–20/hundreds-of-protesters-arrested-in-india-after-water-runs-dry.

Nisbet, E. C., Ostman, R., & Shanahan, J. (2009). Public opinion toward Muslim Americans: Civil liberties and the role of religiosity, ideology, and media use. In A. Sinno (ed.), *Muslims in Western Politics* (pp. 161–99). Bloomington, IN: Indiana University Press.

Ohlsson, L. (2000). *Livelihood Conflicts: Linking Poverty and Environment as Causes of Conflict*. Stockholm: Environmental Policy Unit, Swedish International Development Cooperation Agency.

Ogbu, J. U. (1988). Cultural diversity and human development. *New Directions for Child and Adolescent Development*, *1988*(42), 11–28.

Park, J., Felix, K., & Lee, G. (2007). Implicit attitudes toward Arab-Muslims and the moderating effects of social information. *Basic and Applied Social Psychology*, *29*, 35–45.

Petersen, A. M., Vincent, E. M., & Westerling, A. L. (2019). Discrepancy in scientific authority and media visibility of climate change scientists and contrarians. *Nature Communications*, *10*(1), 1–14.

Pettigrew, T. F. (2021). *Contextual Social Psychology: Reanalyzing Prejudice, Voting, and Intergroup Contact*. Washington, DC: .American Psychological Association.

Pettigrew, T. F. & Tropp, L. M. (2011). *When Groups Meet: The Dynamics of Intergroup Contact*. Philadelphia, PA: Psychology Press.

Petty, R. E. & Cacioppo, J. T. (2012). *Communication and Persuasion: Central and Peripheral Routes to Attitude Change*. New York: Springer-Verlag.

Pew Research Center. (2013). After Boston, little change in views of Islam and violence. www.people-press.org/files/legacy-pdf/5–7–13%20Islam%20Release.pdf.

Downloaded from https://www.cambridge.org/core. Iowa State University Library, on 07 Feb 2022 at 18:20:25, subject to the Cambridge Core terms of use, available at https://www.cambridge.org/core/terms. https://doi.org/10.1017/9781108953078

Pew Research Center. (2015). The American family today. www.pewresearch.org/social-trends/2015/12/17/1-the-american-family-today/.

Pielke, R. (2019). Tracking progress on the economic costs of disasters under the indicators of the sustainable development goals. *Environmental Hazards*, *18*(1), 1–6.

Plante, C., Allen, J. J., & Anderson, C. A. (2017). Likely effects of rapid climate change on violence and conflict. In L. Oglesby (ed.) *The Oxford Research Encyclopedia of Climate Science*. Oxford: Oxford University Press.

Plante, C. & Anderson, C. A. (2017). Global warming and violent behavior. *Association for Psychological Science Observer*, *30*(2), 29–32. www.psychologicalscience.org/observer/global-warming-and-violent-behavior.

Rabin, N. (2018). Understanding secondary immigration enforcement: Immigrant youth and family separation in a border county. *Journal of Law & Education*, *47*(1), 1–40.

Reifman, A. S., Larrick, R. P., & Fein, S. (1991). Temper and temperature on the diamond: The heat-aggression relation in Major League Baseball. *Personality and Social Psychology Bulletin*, *17*, 580–5.

Rettberg, J. W., & Gajjala, R. (2016). Terrorists or cowards: Negative portrayals of male Syrian refugees in social media. *Feminist Media Studies*, *16*(1), 178–81.

Reuters. (2021, April 7). Egypt's Sisi warns of potential for conflict over Ethiopian dam. www.reuters.com/article/us-ethiopia-dam-egypt-sudan/egypts-sisi-warns-of-potential-for-conflict-over-ethiopian-dam-idUSKBN2BU2C3.

Reuveny, R. (2008). Ecomigration and violent conflict: Case studies and public policy implications. *Human Ecology*, *36*, 1–13.

Rivlin, G. (2015). Why the plan to shrink New Orleans failed. *FiveThirtyEight*. https://fivethirtyeight.com/features/why-the-plan-to-shrink-new-orleans-after-katrina-failed/.

Rivlin, G. (2016). White New Orleans has recovered from Hurricane Katrina. Black New Orleans has not. *Talk Poverty*. https://talkpoverty.org/2016/08/29/white-new-orleans-recovered-hurricane-katrina-black-new-orleans-not/.

Rizzo, S. (2019). What's actually in the "Green New Deal" from Democrats? *The Washington Post*. www.washingtonpost.com/gdpr-consent/?next_url=https%3a%2f%2fwww.washingtonpost.com%2fpolitics%2f2019%2f02%2f11%2fwhats-actually-green-new-deal-democrats%2f.

Rotton, J. & Frey, J. (1985). Air pollution, weather, and violent crimes: Concomitant time-series analysis of archival data. *Journal of Personality & Social Psychology*, *49*, 1207–20.

Saeed, A. (2007). Media, racism and Islamophobia: The representation of Islam and Muslims in the media. *Sociology Compass*, *1*, 443–62.

Downloaded from https://www.cambridge.org/core. Iowa State University Library, on 07 Feb 2022 at 18:20:25, subject to the Cambridge Core terms of use, available at https://www.cambridge.org/core/terms. https://doi.org/10.1017/9781108953078

Saleem, M., & Anderson, C. A. (2013). Arabs as terrorists: Effects of stereotypes within violent contexts on attitudes, perceptions and affect. *Psychology of Violence*, *3*, 84–99.

Saleem, M., Prot, S., Anderson, C. A., & Lemieux, A. F. (2017). Exposure to Muslims in media and support for public policies harming Muslims. *Communication Research*, *44*, 841–69.

Saleem, M., Prot, S., Cikara, M. et al. (2015). Cutting Gordian knots: Reducing prejudice through attachment security. *Personality and Social Psychology Bulletin*, *41*, 1560–74.

Sandstrom, H. & Huerta, S. (2013). *The Negative Effects Of Instability On Child Development: A Research Sysnthesis*. Washington, DC: Urban Institute.

Schanzenbach, D. W. & Pitts, A. (2020). How much has food insecurity risen? Evidence from the Census Household Pulse Survey. Institute for Policy Research Rapid Research Report. www.ipr.northwestern.edu/documents/reports/ipr-rapid-researchreports-pulse-hh-data-10-june-2020.pdf.

Scrimshaw, N. S. (1998). Malnutrition, brain development, learning, and behavior. *Nutrition Research*, *18*(2), 351–79.

Sherif, M., Harvey, O. J., White, B. et al. (1954/1961): *Intergroup Conflict and Cooperation: The Robbers Cave Experiment*. Norman, OK: University of Oklahoma, Institute of Intergroup Relations.

Shisanya, C. A. & Khayesi, M. (2007). How is climate change perceived in relation to other socioeconomic and environmental threats in Nairobi, Kenya? *Climatic Change*, *85*(3), 271–84.

Sides, J. & Gross, K. (2013). Stereotypes of Muslims and support for the War on Terror. *Journal of Politics*, *75*, 583–98.

Simister, J. & Cooper, C. (2005). Thermal stress in the U.S.A.: Effects on violence and on employee behavior. *Stress and Health*: *Journal of the International Society for the Investigation of Stress*, *21*, 3–15.

Šisler, V. (2008). Digital Arabs: Representation in video games. *European Journal of Cultural Studies 11*, 203–20.

Smith, H. J., Pettigrew, T. F., Pippin, G. M., & Bialosiewicz, S. (2012). Relative deprivation: A theoretical and meta-analytic review. *Personality and Social Psychology Review*, *16*(3), 203–32.

Spratt, D. and Dunlop, I. (2018). *What Lies Beneath: The Understatement of Existential Climate Risk.* Melbourne, Australia: Breakthrough National Centre for Climate Restoration.

Spratt, D., Dunlop, I., & Barrie, A. C. (2019). *Existential climate-related security risk.* Melbourne, Australia: Breakthrough National Centre for Climate Restoration..

Downloaded from https://www.cambridge.org/core. Iowa State University Library, on 07 Feb 2022 at 18:20:25, subject to the Cambridge Core terms of use, available at https://www.cambridge.org/core/terms. https://doi.org/10.1017/9781108953078

Stephan, W. G., Ybarra, O., & Rios Morrison, K. (2009). Intergroup threat theory. In T. Nelson (ed.), *Handbook of Prejudice* (pp. 43–59). Mahwah, NJ: Lawrence Erlbaum Associates.

Sui, S. X., Ridding, M. C., & Hordacre, B. (2020). Obesity is associated with reduced plasticity of the human motor cortex. *Brain Sciences*, *10*(9), 579.

Tajfel, H. (1981). *Human Groups and Social Categories: Studies in Social Psychology*. Cambridge: Cambridge University Press.

Tomlinson, H. (2019, June 25). Police guard water tankers from rioters as India buckles under 50 C heatwave. *The Times*. www.thetimes.co.uk/article/indias-heatwave-causes-death-drought-and-despair-mbvrns7r2.

Tukachinsky, R., Mastro, D., & Yarchi, M. (2015). Documenting portrayals of race/ethnicity on primetime television over a 20-year span and their association with national-level racial/ethnic attitudes. *Journal of Social Issues*, *71*, 17–38.

United States Department of Agriculture. (2020). Food security in the U.S. USDA – Economic Research Service. www.ers.usda.gov/topics/food-nutrition-assistance/food-security-in-the-us/key-statistics-graphics.aspx.

Uskul, A. K. & Cross, S. E. (2020). Socio-ecological roots of cultures of honor. *Current Opinion in Psychology*, *32*, 177–80.

Valentino, N. A. (1999). Crime news and the priming of racial attitudes during evaluations of the president. *Public Opinion Quarterly*, 293–320.

Van de Vliert, E. (2009). *Climate, affluence, and culture*. New York: Cambridge University Press.

Van Lange, P. A., Rinderu, M. I., & Bushman, B. J. (2017). Aggression and violence around the world: A model of climate, aggression, and self-control in humans (CLASH). *Behavioral and Brain Sciences*, *40*(75). www.cambridge.org/core/journals/behavioral-and-brain-sciences/article/abs/aggression-and-violence-around-the-world-a-model-of-climate-aggression-and-selfcontrol-in-humans-clash/39F8C1E903B0A355948316C3B9003740.

Van de Vliert, E. (2013). Climato-economic habitats support patterns of human needs, stresses, and freedoms. *Behavioral and Brain Sciences*, *36*(5), 465–80.

Van de Vliert, E., Schwartz, S. H., Huismans, S. E., Hofstede, G., & Daan, S. (1999). Temperature, cultural masculinity, and domestic political violence: A cross-national study. *Journal of Cross-Cultural Psychology*, *30*, 291–314.

Vazsonyi, A. T., Flannery, D. J., & DeLisi, M. (eds.) (2018). *The Cambridge Handbook of Violent Behavior and Aggression*. Cambridge: Cambridge University Press.

Vrij, A., van der Steen, J., & Koppelaar, L. (1994). Aggression of police officers as a function of temperature: An experiment with the Fire Arms Training System. *Journal of Community and Applied Social Psychology*, *4*, 365–70.

Downloaded from https://www.cambridge.org/core. Iowa State University Library, on 07 Feb 2022 at 18:20:25, subject to the Cambridge Core terms of use, available at https://www.cambridge.org/core/terms. https://doi.org/10.1017/9781108953078

Walter, E. J. & Carraretto, M. (2016). The neurological and cognitive consequences of hyperthermia. *Critical Care*, *20*(1), 1–8.

Warburton, W. A. & Anderson, C. A. (2018). Aggression. In V. Zeigler-Hill & T. K. Shackelford (eds.), *The SAGE Handbook of Personality and Individual Differences: Applications of Personality and Individual Differences* (pp. 183–211). Thousand Oaks, CA: Sage.

Wariaro, V. & Hoopert, D. (2018). Global catastrophic risks 2018. Stockholm, Global Challenges Foundation, 24.

Webster, B. (2018). BBC freezes out climate sceptics. *The Times*. www.thetimes.co.uk/edition/news/bbc-freezes-out-climate-sceptics-fqhqmrfs6.

Wilkowski, B. M., Meier, B. P., Robinson, M. D., Carter, M. S., & Feltman, R. (2009). "Hotheaded" is more than an expression: The embodied representation of anger in terms of heat. *Journal of Personality and Social Psychology*, *9*, 464–77.

Wilson, K. R., Hansen, D. J., & Li, M. (2011). The traumatic stress response in child maltreatment and resultant neuropsychological effects. *Aggression and Violent Behavior*, *16*(2), 87–97.

Wittbrodt, M. T., Sawka, M. N., Mizelle, J. C., Wheaton, L. A., & Millard Stafford, M. L. (2018). Exercise heat stress with and without water replacement alters brain structures and impairs visuomotor performance. *Physiological Reports*, *6*(16), e13805.

Yamaguchi, T., Shimizu, K., Kokubu, Y. et al. (2019). Effect of heat stress on blood-brain barrier integrity in iPS cell-derived microvascular endothelial cell models. *PloS one*, *14*(9), e0222113.

Yaqub, B. A. (1987). Neurologic manifestations of heatstroke at the Mecca pilgrimage. *Neurology*, *37*(6), 1004.

Yasayko, J. (2010). Attacks on transit drivers as a function of ambient temperature. Master's thesis, Simon Fraser University.

Zhang, D. D., Brecke, P., Lee, H. F., He, Y. O., & Zhang, J. (2007). Global climate change, war, and population decline in recent human history. *Proceedings of the National Academy of Science*, *104*, 19214–19.

Zhang, D. D., Zhang, J., Lee, H. F., & He, Y. O. (2007). Climate change and war frequency in eastern china over the last millennium. *Human Ecology*, *35*, 403–14.

Zillmann, D. (1978). Attribution and misattribution of excitatory reactions. In J. Harvey, W. Ickes, & R. Kidd (eds.), *New Directions in Attribution Aesearch* (vol. 2, pp. 335–68). Hillsdale, NJ: Erlbaum.

Downloaded from https://www.cambridge.org/core. Iowa State University Library, on 07 Feb 2022 at 18:20:25, subject to the Cambridge Core terms of use, available at https://www.cambridge.org/core/terms. https://doi.org/10.1017/9781108953078

Downloaded from https://www.cambridge.org/core. Iowa State University Library, on 07 Feb 2022 at 18:20:25, subject to the Cambridge Core terms of use, available at https://www.cambridge.org/core/terms. https://doi.org/10.1017/9781108953078

Cambridge Elements

Applied Social Psychology

Susan Clayton
College of Wooster, Ohio
Susan Clayton is a social psychologist at the College of Wooster in Wooster, Ohio. Her research focuses on the human relationship with nature, how it is socially constructed, and how it can be utilized to promote environmental concern.

Editorial Board

Dominic Abrams *University of Kent*
Heather Bullock *University of California-Santa Cruz*
Amanda Diekman *Indiana University – Bloomington*
Immo Fritsche *Leipzig University*
Taciano Milfont *Victoria University of Wellington*
Lou Penner *Wayne State University*
Michael Platow *Australia National University Canberra*
Peggy Stockdale *Indiana University–Purdue University, Indianapolis*
Janet Swim *Pennsylvania State University*

About the Series

Many social psychologists have used their research to understand and address pressing social issues, from poverty and prejudice to work and health. Each Element in this series reviews a particular area of applied social psychology. Elements will also discuss applications of the research findings and describe directions for future study.

Downloaded from https://www.cambridge.org/core. Iowa State University Library, on 07 Feb 2022 at 18:20:25, subject to the Cambridge Core terms of use, available at https://www.cambridge.org/core/terms. https://doi.org/10.1017/9781108953078

Cambridge Elements

Applied Social Psychology

Elements in the Series

Empathy and Concern with Negative Evaluation in Intergroup Relations: Implications for Designing Effective Interventions
Jacquie D. Vorauer

The Psychology of Climate Change Adaptation
Anne van Valkengoed and Linda Steg

Undoing the Gender Binary
Charlotte Chucky Tate, Ella Ben Hagai, and Faye J. Crosby

Selves as Solutions to Social Inequalities: Why Engaging the Full Complexity of Social Identities is Critical to Addressing Disparities
Tiffany N. Brannon, Peter H. Fisher, and Abigail J. Greydanus

Identity Development During STEM Integration for Underrepresented Minority Students
Sophie L. Kuchynka, Alexander E. Gates, and Luis M. Rivera

The Psychology of Effective Activism
Robyn Gulliver, Susilo Wibisono, Kelly S. Fielding, and Winnifred R. Louis

Learning from Video Games (and Everything Else): The General Learning Model
Douglas A. Gentile and J. Ronald Gentile

Climate Change and Human Behavior: Impacts of a Rapidly Changing Climate on Human Aggression and Violence
Andreas Miles-Novelo and Craig A. Anderson

A full series listing is available at: www.cambridge.org/EASP

Downloaded from https://www.cambridge.org/core. Iowa State University Library, on 07 Feb 2022 at 18:20:25, subject to the Cambridge Core terms of use, available at https://www.cambridge.org/core/terms. https://doi.org/10.1017/9781108953078
